# Environmental correlates of phenotypic evolution in ecologically diverse *Liolaemus* lizards

**DOI:** 10.1002/ece3.9009

**Published:** 2022-06-16

**Authors:** Danielle L. Edwards, Luciano J. Avila, Lorena Martinez, Jack W. Sites, Mariana Morando

**Affiliations:** ^1^ The Department of Life & Environmental Sciences University of California Merced California USA; ^2^ Instituto Patagónico para el Estudio de los Ecosistemas Continentales (IPEEC‐CONICET) Puerto Madryn Argentina; ^3^ Vigo Spain; ^4^ Department of Biology and M.L. Bean Life Science Museum Brigham Young University (BYU) Provo Utah USA; ^5^ Trenton Kentucky USA

**Keywords:** ecomorphology, environmental variation, *Liolaemus gracilis* species complex, morphological evolution, phylogenetic comparative methods

## Abstract

Evolutionary correlations between phenotypic and environmental traits characterize adaptive radiations. However, the lizard genus *Liolaemus*, one of the most ecologically diverse terrestrial vertebrate radiations on earth, has so far shown limited or mixed evidence of adaptive diversification in phenotype. Restricted use of comprehensive environmental data, incomplete taxonomic representation and not considering phylogenetic uncertainty may have led to contradictory evidence. We compiled a 26‐taxon dataset for the *Liolaemus gracilis* species group, representing much of the ecological diversity represented within *Liolaemus* and used environmental data to characterize how environments occupied by species' relate to phenotypic evolution. Our analyses, explicitly accounting for phylogenetic uncertainty, suggest diversification in phenotypic traits toward the present, with body shape evolution rapidly evolving in this group. Body shape evolution correlates with the occupation of different structural habitats indicated by vegetation axes suggesting species have adapted for maximal locomotory performance in these habitats. Our results also imply that the effects of phylogenetic uncertainty and model misspecification may be more extensive on univariate, relative to multivariate analyses of evolutionary correlations, which is an important consideration in analyzing data from rapidly radiating adaptive radiations.

## Graphical abstract

We used environmental data to characterize how environments occupied by Liolaemus gracilis complex species’ relate to phenotypic evolution. Our analyses suggest diversification in phenotypic traits, especially body shape evolution which correlates with the occupation of different structural habitats. We also show that the effects of phylogenetic uncertainty and model misspecification may be more extensive on univariate, relative to multivariate analyses of evolutionary correlations.

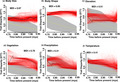

## 1 INTRODUCTION

Understanding how phenotypic diversification relates to ecological variation forms a major component of evolutionary biology (Endler, 1977; Mayr, 1942), particularly the study of adaptive radiation. Adaptive radiation represents rapid diversification coupled with adaptation to different ecological niches that have culminated in some of the most diverse clades on earth (Gavrilets & Losos, 2009; Grant & Grant, 2008; Schluter, 2009; Yoder et al., 2010) and spawned major fields in evolutionary biology investigating the association of adaptive variation to speciation rates (Marques et al., 2019). Phenotype–environment associations, or covariation between species or population phenotypes and environmental variables, often result from the evolution of divergent functional capabilities for optimal performance in specific ecological contexts (i.e., Arnold, 1983). Therefore, revealing the evolutionary association between environmental and phenotypic traits (i.e., Irschick et al., 2008) is important for understanding the process of adaptive radiation (Gavrilets, 2009; Glor, 2010; Harder & Schluter, 2001).

Studies linking environmental variables to phenotypic traits have exploded in recent years, especially given advancements in geometric morphometrics (e.g., Bastir et al., 2019; Klingenberg, 2010), CT scanning (i.e., Gignac et al., 2016), and multivariate evolutionary analyses (Adams & Collyer, 2019; Clavel et al., 2019; Clavel & Morlon, 2020). Nevertheless, to date, because of the ease of obtaining data, most studies have largely been limited to studying climatic variables (namely average temperatures and precipitation) and their impacts on body size evolution. However, the climate is only one aspect of an organism's functional environment which may have limited impacts on organism performance, and body size represents a limited perspective on phenotypic evolution (Wainwright, 2007). Furthermore, various aspects of an organism's environment may impose selection in different ways on alternate elements of the phenotype (Irschick et al., 2008). Such variation likely has led to inconsistency in the results of studies that have assessed phenotypic–environment correlations in various groups. On top of largely consisting of tests of evolution restricted to univariate dependent variables (namely body size) relative to climate, many macroevolutionary studies have also had limited exploration of the impacts of phylogenetic uncertainty. This is problematic given the amount of research indicating that rapidly evolving clades also result in a large amount of discordance in phylogenetic estimation and hybridization—leading to uncertainty in reconstructing topological relationships and timing of divergences (Ortego & Knowles, 2022; Rangel et al., 2015; Talavera et al., 2013).

Squamate reptiles display a startling array of phenotypic and ecological diversity, but with inconsistent results with respect to the impact of the environment on phenotypic evolution depending on the phenotypic dependent variable and the environmental correlate tested. Multiple squamate families display strong evolutionary relationships between body shape (i.e., limb and body dimensions) and structural habitat characteristics (Losos, 1990; Melville & Swain, 2000; Tulli et al., 2009; Wiens et al., 2006), providing evidence for the evolution of optimal locomotor performance for different microhabitat characteristics and not climate‐driven evolution. Alternatively, body size variation, an important ecomorphological axis of variation in snakes, is not correlated with climate in Lampropeltine snakes (Pyron & Burbrink, 2009) and in general squamates show inconsistent patterns of body size evolution in response to the occupation of different climatic regimes (Slavenko et al., 2019). It may be that body size evolution is not that prominent in many squamate clades, and that shape variation is a more important axis of phenotypic evolution that is predicted by structural habitat characteristics, rather than climate. However, shape variation in invasive *Anolis* lizards is not consistent with structural habitat differences, and rapid adaptation, between native and invasive populations (Kolbe et al., 2007) indicating that environment does not always lead to rapid morphological change even in adaptive radiations. Thus, the exploration of evolutionary relationships between multiple phenotypic and environmental axes is essential to our understanding of how adaptive evolution progresses and what factors are the most important in aiding the diversification of rapidly radiating groups.

The lizard genus *Liolaemus* occupies one of the widest climate ranges observed in a vertebrate clade, extending from sea level on the southern Pacific and Atlantic coasts, through the Atacama Desert, Validivian Forest, cold Patagonian steppes, to the high Andes (~5000 m elevation; (Cei, 1986; Donoso‐Barros, 1966; Espinoza et al., 2004; Schulte et al., 2000). Given that the 260 currently described species of *Liolaemus* (Uetz, 2019) cover such ecological breadth, hypotheses abound regarding the evolutionary mechanisms by which its species diversity arises. Speciation within *Liolaemus* has been characterized by a slow, but consistent, accumulation of lineages with significant diversification in body size (Harmon et al., 2003; but see Olave et al., 2020). Body size has also been shown to covary with ecological, life history, and other morphological traits, like ovipary/viviparity and altitude in *Liolaemus* (Meiri, 2008; Pincheira‐Donoso et al., 2011). Harmon et al. (2003) suggested that convergent body size evolution among sub‐clades had occurred in response to the occupation of different ecophysiological environments across elevational gradients. Espinoza et al. (2004) further showed that smaller animals resided in cooler climates, and hypothesized that the evolution of herbivory and thermal constraints limited the evolution of body size. Body size was shown to increase with latitude, relating to thermal amplitude and temperature, in the *Liolaemus geotschi* species complex, supporting the notion that variation in body size relates to the thermal biology of *Liolaemus* species in different climatic regimes (Azócar et al., 2013). The occupation of cooler climates has also been associated with the evolution of viviparity in *Liolaemus* (Esquerré et al., 2019; Pincheira‐Donoso et al., 2013).

In contrast, traits like body shape and limb proportions may not evolve in response to habitat diversification within *Liolaemus* (Jaksić et al., 1980; Schulte et al., 2004; Tulli et al., 2012). However, this is not consistent and studies have also largely focused on broad evolutionary patterns across a sparsely sampled *Liolaemus* phylogeny. Such limited taxon sampling may impede meaningful analyses of evolutionary patterns (e.g., Heath et al., 2008), particularly the detection of evolutionary correlations between ecology and morphology (e.g., Ackerly, 2000). Analyses of macroevolutionary trends in *Liolaemus* have largely been undertaken without assessing the impacts of phylogenetic uncertainty, which is problematic in a group that has experienced extensive hybridization and rapid radiation (Esquerré et al., 2021; Olave et al., 2018). In support of environmentally driven morphological shape change, Tulli et al. (2009)) suggested that limb morphology in *Liolaemus* evolved for maximal performance in different habitats (Tulli et al., 2011), especially in response to substrate type (Tulli et al., 2016). Foot morphology may also be evolutionary labile in *Liolaemus* (Tulli et al., 2012). Narrower pelvises and longer tibias and metatarsals (i.e., foot length) have been connected with greater sprint speeds in lizards (Irschick & Jayne, 2000), especially in phrynosomatids which have similar morphologies to *Liolaemus* (Bergmann & Irschick, 2010). Furthermore, foot and limb morphology covaries with escape behavior in *Liolaemus* (Schulte et al., 2004).

We use multivariate comparative phylogenetic analyses to test the relationship between body size and shape evolution and multiple classes of environmental data in the well‐sampled and ecologically diverse *L. gracilis* group and allied taxa. This clade includes up to 33 candidate species, including described and undescribed species. The distribution crosses the cold Patagonian steppe deserts (i.e., *Liolaemus bibronii* sensu stricto), mixed deciduous forests (i.e., *Liolaemus vhagar*), and highland steppe biomes (i.e., *Liolaemus puna*), and thus represents much of the environmental diversity encompassed by the genus (Abdala et al., 2015; Morando et al., 2007; Olave et al., 2011). This is the first analysis of multivariate morphological evolution patterns at this phylogenetic scale within *Liolaemus*, with extensive taxon sampling. We conduct all our analyses by explicitly incorporating phylogenetic uncertainty into our interpretation of results. Specifically, we assess the diversification of body size and shape, along with vegetation, temperature, precipitation, and elevation data, test if environmental and morphological evolution are evolutionarily correlated, and discuss how patterns of trait evolution within this *Liolaemus* clade compare with those inferred across *Liolaemus* as a whole, and more broadly in squamates. We also discuss the impact of model choice and phylogenetic uncertainty where evolutionary correlation assesses phenotype–environment correlations with dependent multivariate and univariate data.

## 2 MATERIALS AND METHODS

### 2.1 Taxon sampling

There are currently 33 species considered within the *L. gracilis* species complex (Quinteros et al., 2020), including 25 described and 8 candidate species. Recent taxonomic papers have addressed the phylogenetic history and taxonomic composition of this species complex, but there are caveats. Portelli and Quinteros (2018) suggested some supported groups were not congruent with those Quinteros et al. (2020) propose. Also, cases of hybridization with phylogenetically distantly related species confuse taxonomy (e.g. *Liolaemus abdalai* may not be part of the *L. gracilis* complex; Morando and Avila (2020)). Within the *L. gracilis* complex, there are two clades (Portelli & Quinteros, 2018), one mostly central‐northern clade, the *Liolaemus robertmertensi* clade and a mostly southern clade, the *L. bibronii* clade. Our taxon sampling includes representatives of both these clades, with additional candidate species that Esquerré et al. (2021) show is monophyletic.

**FIGURE 1:**
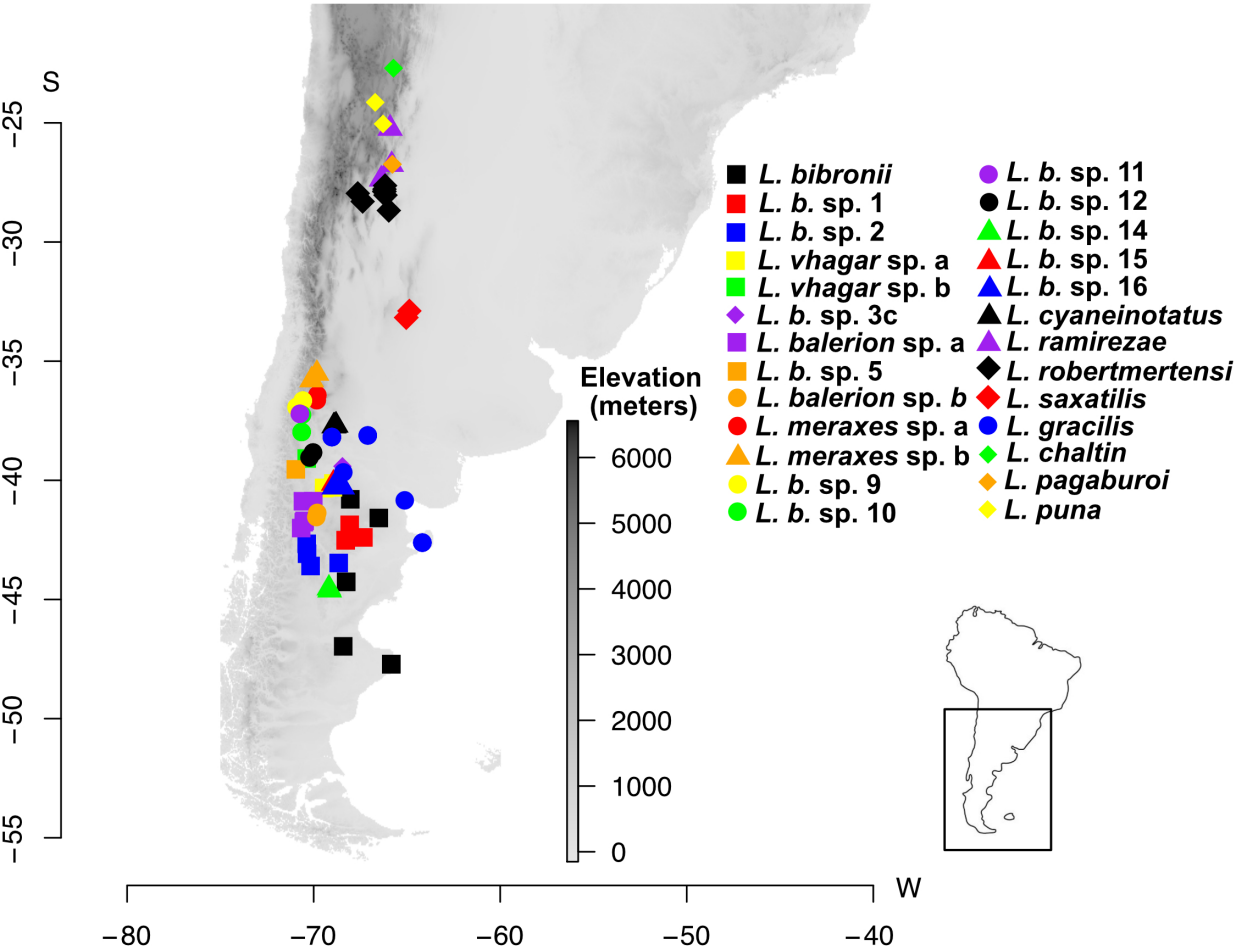
Distribution map of the *L. gracilis* species complex shown overlain over a digital elevation model of Patagonia, position relative to South America is shown inset. Different species are indicated by the various colored and shaped symbols as outlined in the inset key

We sampled tissues from 47 individuals across 26 terminal taxa of the *L. gracilis* species complex (Table S1; Figure 1). Several candidate species, assigned to *L. bibronii*, including clades 1–2, 3c, 5, 9–12, and 14–16, are novel to this paper. These novel candidate species were delimited using methods outlined in Wiens and Penkrot (2002) using tree‐based methods with morphological data (Martinez, 2012). Our sampling design covers most of the known diversity within this complex, and includes most of the described species (*Liolaemus chaltin, Liolaemus pagaburoi, Liolaemus cyaneinotatus, Liolaemus bibronii, L. gracilis*, *Liolaemus ramirezae*, *L. robertmertensi*, *L. puna, Liolaemus balerion, Liolaemus meraxes, Liolaemus Vhagar*, and *Liolaemus. saxatilis*). Several candidate species were included in our molecular dataset for reconstructing phylogenetic relationships but were dropped from analyses due to a lack of morphological data (Table S1), this included *L. bibronii* clades 6, 17, and 19 (Morando et al., 2007). Outgroup taxa, *Liolaemus walkeri* and *Liolaemus punmahuida*, were included in phylogenetic reconstruction, but not included in comparative analyses.

### 2.2 Genetic data

DNA was extracted using Qiagen DNeasy® tissue kit following the protocol provided by the manufacturer. Two mitochondrial fragments were sequenced, cytochrome‐b (cyt‐b (725 base pairs); (Palumbi, 1996; Whiting et al., 2003) and 12S (883 base pairs) (Wiens et al., 2010), and four anonymous nuclear loci, LB9C (740 base pairs), LPB4G (661 base pairs), LPA11E (785 base pairs) (Olave et al., 2011), and LPB11E (823 base pairs; this study). See Table S2 for details of each marker. Methods for marker development followed those outlined by Morando et al. (2014). PCR and sequencing protocols followed Morando et al. (2003), Morando et al. (2004) and Noonan and Yoder (2009) for mitochondrial and nuclear fragments, respectively. Sequencher v4.10. (Gene Codes Corporation Inc.™ 2007) was used to edit and align all the fragments, and alignments were verified by eye.

### 2.3 Environmental data

Vegetation data are from satellite data taken from the NASA MODIS/Terra database (https://modis.gsfc.nasa.gov/data/). For measures of vegetation structure, we used mean normalized difference vegetation index (NDVI; a measure of vegetative biomass) and standard error of NDVI (describing seasonality in NDVI), both data from MODIS product MODIS13A1 (see https://lpdaac.usgs.gov/products/mod13a1v006/), and proportional tree cover, using MODIS product MODIS44b (see https://lpdaac.usgs.gov/products/mod44bv006/). MODIS data from the years 2002–2011 were downloaded using the *ModisDownload.R* source code v3.2, now part of the *rts* package v1.1.3 (Naimi, 2021). Datasets (HDF files) were converted to TIFF files using the MRT (Modis Reprojection Tool) Batch Reprojection java tool, *MRTBatch.jar*. Tree cover data are collected annually, so we were not able to calculate anything other than an average across the 10‐year period using the *calc* function of the *raster* package v3.5.11 (Hijmans, 2021). For NDVI, data are collected at 16‐day intervals. First, we calculated mean annual variables, then averaged these annual subsets of data across the 10‐year period using the *calc* function, as above for tree cover, for mean NDVI. Then we calculated yearly standard deviations from the 16‐day data and averaged these across the 10‐year period using the *calc* function, to represent average seasonal fluctuation in vegetative biomass.

Vegetation variables were chosen to reflect structural vegetation features of the habitat. NDVI measures the greenness of vegetation and provides an index of vegetative productivity and biomass (Fensholt et al., 2004) and for our purposes provides a measure of the productivity of habitats—which is possibly linked to phenotypic evolution. Climate data were obtained from publicly available GIS layers from the WorldClim Database (http://www.worldclim.org; Hijmans et al., 2005). Temperature variables included WorldClim layers 1–11, while precipitation variables included WorldClim layers 12–19. Climate and vegetation data were extracted for each individual sample using the spatial coordinates associated with morphological specimens. All variables were log‐transformed prior to analysis. For worclim variable B4 (Temperature Seasonality), values were divided by 100 prior to log transformation given these are expressed as percentages. For worldclim variables B6 (Minimum Temperature of the Coldest Month) and B14 (Precipitation of the Driest Month), values of 100 and 1, respectively were added to ensure positive values prior to log transformation. Datasets considered here comprised of related variables measures in similar scales for temperature, precipitation, vegetation, and elevation.

### 2.4 Phenotypic data

Phenotypic data were collected by one of the authors from specimens utilized in genetic analyses where possible (135 specimens; 5.7 ± 4.3 [mean ± standard deviation] individuals per species; Table S1). Specimens are located in the LJAMM Herpetological Collection at IPEEC‐CONICET, the Fund Miguel Lillo Collection (FML), The American Museum of Natural History Collection (AMNH), the Museum of Biodiversity San Diego State Collection at The Museum of Vertebrate Zoology UC Berkeley (SDSU), Museo Universidad San Marcos Collection (MUSM), the Monte L. Bean Life Science Museum Collection at Brigham Young University (BYU), and a single specimen from Robert Espinoza's Field Collection (REE). Only males were used for this data set to avoid complications of sexual dimorphism in characters. Measurements of snout‐vent length (SVL; mm) were collected as a proxy for body size. Morphological measurements collected were axilla‐groin length (AG), arm length (AL), tibia length (TbL), foot length (FL), and pelvic girdle width (PW; all in mm). Measurements were missing for *L. bibronii* candidate species 5 for tibia length only, therefore for completeness values were imputed using a regression approach using the relationship between body size and tibia length to predict the missing tibia value from body size information available for that candidate species. Regression analyses were performed using the *lm* function of the *stats* package v4.0.2 (R Core Team, 2020).

### 2.5 Ecological and phenotypic trait data reduction for comparative analyses

Prior to analysis, all morphological variables were standardized via log‐transformation. Body shape in lizards is connected to locomotory performance (Bergmann & Irschick, 2010; Irschick & Jayne, 2000), is labile in *Liolaemus* (Tulli et al., 2012) and connected to escape behavior (Schulte et al., 2004). To allow for more functional analyses of the evolution of shape in relation to performance, by allowing for the inclusion of allometry (Bergmann & Irschick, 2010), we corrected for the effects of body size on shape variables by calculating the ratios of AG, AL, TbL, FL, and PW with SVL (e.g., AG:SVL). All morphological variables displayed significant regressions against SVL validating the use of ratios (Table S3). Given the potential for differences in patterns of evolution, and in evolutionary correlations between shape, size, and environmental variables (vegetation, precipitation, temperature, and elevation), several separate datasets were analyzed. For size, shape, temperature, precipitation, and vegetation data variables were averaged for each species. To simplify environmental datasets containing potentially correlated variables, each dataset underwent variable reduction prior to further analyses using principle components analyses (PCA) implemented in the *dudi.pca* R function of the *vegan* package v2.5–7 (Oksanen et al., 2020). Temperature, precipitation, and vegetation axes comprising 99% of the variation were retained for further analysis. Output from these analyses, including variable loadings, are in Table S4. Variations in datasets across the phylogeny can be found in Figures S1–S5. Univariate datasets were size (SVL) and elevation, multivariate datasets were shape, vegetation, precipitation, and temperature.

### 2.6 Time‐calibrated species tree analyses

We used *BEAST v1.8.2 to estimate the species tree and lineage divergence times simultaneously (Heled & Drummond, 2010). Lineage divergence times were estimated using calibration methods described in (McCormack et al., 2011), as no internal fossil calibrations exist for the *L. gracilis* species group. Species tree analyses were time‐calibrated using a mean rate of evolution for the cytochrome‐b locus (0.0193555 mutation rate/site/million years) calculated by Olave et al. (2015) using a fossil that places the divergence between *Eulaemus* and *Liolaemus* at 20 million years ago (Albino, 2008). This rate was fixed for the cytochrome‐b locus only in our species tree analyses under a strict clock model (McCormack et al., 2011) using multilocus genetic data in *BEAST (Heled & Drummond, 2010) for 500 million generations, sampling every 5000 steps with a 10% burnin. Tree files for each run were combined and summarized after confirming singular run convergence (i.e., high ESS values), topological convergence across runs, and removing burnin using Tracer v1.7 (Rambaut et al., 2018). The median time‐calibrated species tree was then used in all further comparative analyses, along with 1000 trees randomly sampled from the posterior distribution where appropriate.

### 2.7 Patterns of ecological and phenotypic diversification

Disparity‐through‐time (DTT) analyses (Harmon et al., 2003) were used to assess changes in subclade disparity in phenotypic and environmental traits throughout the evolutionary history of the *L. gracilis* group. The disparity is calculated as the average pairwise Euclidean distance between species (MDI). To provide a temporal context to trait change, the observed disparity is calculated as the average relative disparity at each node, including all subclades present at that particular time (Harmon et al., 2003). Values of MDI closer to 1 indicate that species within subclades overlap substantially, having independently evolved to occupy similar convergent regions of trait space. MDI values that are closer to 0 suggest, alternatively, that variation is distributed among subclades (Harmon et al., 2003). Some have suggested that this latter case, when lower than the null model of Brownian Motion (BM) evolution, represents constrained trait evolution (Smith et al., 2011). These analyses rely on comparing observed data to a BM null model on slow, constant change through time.

DTT analyses were undertaken using means of all variables for multivariate datasets and for univariate datasets. Analyses were performed using the *dtt* function of the *geiger* R package v2.0.7 (Pennell et al., 2014) with the effects of phylogenetic uncertainty mapped using the methods outlined in Edwards et al. (2015), for iterating DTT analyses over 1000 trees randomly drawn from the posterior distribution of species trees. We incorporate phylogenetic uncertainty because posterior trees can vary in not only the topological relationships inferred but also the timing of divergencies among species. Therefore, testing many trees from the posterior allows us to incorporate uncertainty in topological reconstruction and divergence timing in interpreting our results. This analysis allows for an interpretation of observed subclade disparity in light of phylogenetic uncertainty, both regarding the magnitude of observed subclade disparity relative to the null and the timing of shifts throughout history. The magnitude of difference between observed disparity and the null model can be used to determine if trait evolution constrained (below the BM null), or if traits are have diversified at any point in history (above the null) among subclades (e.g., Smith et al., 2011). For instance, traits typically showing an early burst model of evolution will show increased disparity early in evolution followed by declining to present relative to the BM null model.

### 2.8 Mode of environmental and phenotypic trait evolution

Because there could be differences in evolutionary trajectories between clades (Uyeda et al., 2018) driving evolutionary correlation, we sought to test for these differences in a phylogenetic framework using MANOVA and ANOVA analyses for multivariate and univariate data respectively. These analyses rely on deciphering the appropriate model of trait evolution first which DTT analyses suggest might not always be BM. To determine the model of trait evolution that best fit each dataset, we compared BM, Ornstein–Uhlenbeck (OU), and Early Burst (EB) models of trait evolution for both multivariate and univariate trait datasets. We assessed model fit using the *mvBM, mvOU*, and *mvEB* functions of the *mvMORPH* package in R v1.1.4 (Clavel et al., 2015). For these analyses, we used the “rpf” method to impose a computationally intensive generalized least squares approach; iterating over 1000 trees from the posterior distribution. For each tree, we used the AICc score weights to assess which model of trait evolution best fit the data using the *aicw* function of the *mvMORPH* package v1.1.4 (Clavel et al., 2015).

To test if there were differences in environmental and phenotypic traits that were largely driven by a northern and southern lineage split, we assessed if there were significant differences between these groups in trait values. For multivariate trait datasets, we used a phylogenetic MANOVA implemented in the *mvgls* function setting model to the model of best fit chosen (see above), error to “true” to allow for estimation of measurement error from the data, a method to “LOOCV” for leave one out cross‐validation of the penalized log‐likelihood, penalty to “RidgeArch” to linearly shrink the covariance structure to the target of “unitVariance” which is proportional diagonal variance relative to identity. Posthoc tests were undertaken using the *manova.gls* function with 999 permutations and the Wilks test statistic. Both *mvgls* and *manova.gls* are part of the *mvMORPH* package v1.1.4 (Clavel et al., 2015). Wilks statistic is equivalent to an *F‐*statistic, which we calculated for univariate analyses, making comparisons possible. For univariate trait analyses (i.e., elevation), we undertook a phylogenetic ANOVA using *gls* from the package *nlme* v3.1–152 (Pinheiro et al., 2013) and undertook an ANOVA using the *anova* function of the base R package v4.0.2. All ANOVA/MANOVA analyses were iterated over 1000 trees from the posterior distribution to account for phylogenetic uncertainty. Analyses were implemented using the model of trait evolution that best fit the data.

### 2.9 The evolutionary relationships between environment and phenotype

Significant evolutionary relationships between environmental and phenotypic traits typify ecologically driven phenotypic evolution (Garland Jr. et al., 1992; Glor, 2010). To investigate how environmental variables explain variance in phenotypic trait values, we investigated evolutionary correlations using phylogenetic linear models to understand the relationships between phenotype and environment. Evolutionary correlation tests were undertaken using the *mvgls* function of the *mvMORPH* package v.1.1.4 (Clavel et al., 2015). The evolutionary model of best fit, between the BM, OU, and EB models, was determined using *mvgls* function setting error to “TRUE” and using the “LL” method and iterated across 1000 randomly chosen posterior trees. The AIC values of respective models were then compared for each individual tree and the model that fit the majority of trees was selected as the best fit using the *aicw* function. The evolutionary correlation was then estimated using the same parameter settings model testing analyses. Univariate data were analyzed in a similar manner using the *gls* function of the *nlme* package v3.1.153 (Pinheiro et al., 2013) and using the *corBrownian*, *corMartins*, and *corBlomberg* correlation structures from the *ape* r package v5.6–1 (Paradis & Schliep, 2019) for the BM, OU, and EB models respectively. To ensure we were testing an early burst, and not a decelerating model for the *corBlomberg* structure, values of g were fixed at 0.5.

Both univariate and multivariate analyses were undertaken using the best‐selected model of trait evolution identified for the correlation structure between compared datasets. Analyses were iterated over 1000 randomly chosen trees, randomly chosen from the posterior distribution of trees to account for phylogenetic uncertainty. Dependent variables consisted of the PC scores of multivariate datasets (temperature, precipitaiton, and vegetation) or univariate data (elevation), analyzed separately. From this analysis of phylogenetic uncertainty, we calculated the proportion of trees supporting relationships between dependent and predictor variables, and the mean and 95% CI of test statistics and model coefficients.

## 3 RESULTS

### 3.1 Phylogenetic relationships and timing of diversification

**FIGURE 2:**
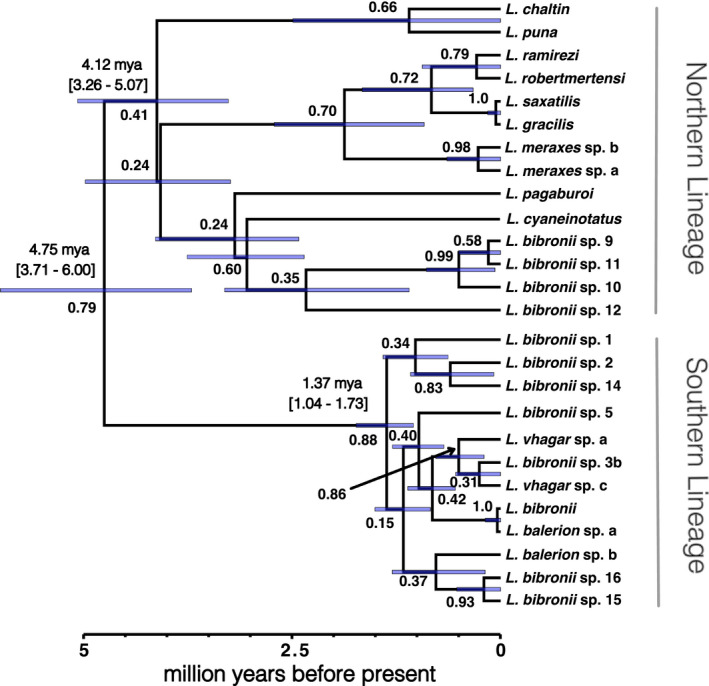
Median time‐calibrated species tree showing the evolutionary relationships among species within the *Liolaemus gracilis* species complex, including a predominantly southern and northern clade. The 95% confidence intervals on divergence dates are shown in the gray node bars, and as the text above the major nodes for the northern, southern, and the most recent common ancestor of the group. A time axis is also displayed below the tree. Numbers around nodes represent the posterior probabilities for species tree support for each node

Time‐calibrated phylogenetic analyses show an origin for the *L. gracilis* group ~3.7–6 million years ago (Figure 2). Posterior support for relationships among species ranges from low to high, with no clear patterns of topological uncertainty concentrated in specific parts of the tree. Our phylogenetic analyses recover main clades within the broader *L. gracilis* group. A northern lineage (Figures 1 & 2) contains most currently described species (*L. saxatilis, L. ramirezae, L. gracilis, L. robertmertensi, L. cyaneinotatus, L. puna, L. chaltin*, and *L. pagaburoi*) as well as several candidate species currently assigned to *L. bibronii* and corresponds to the *L. robertmertensi* group of Quinteros et al. (2020). This northern lineage arose between 3.3 and 5 million years ago, and displays species divergences ranging from early divergence through to the present. Alternatively, the lineage containing most southern species (Figures 1 & 2), including *L bibronii* sensu stricto and many other lineages assigned to *L. bibronii*, termed the *L. bibronii* group of Quinteros et al. (2020), has a more recent common ancestor arising 1–2 million years ago, and shows a subsequent recent and rapid divergence of lineages and species throughout southern Patagonia. We refer to these clades as the northern and southern lineages respectively throughout.

### 3.2 Patterns of ecological and phenotypic diversification

**FIGURE 3:**
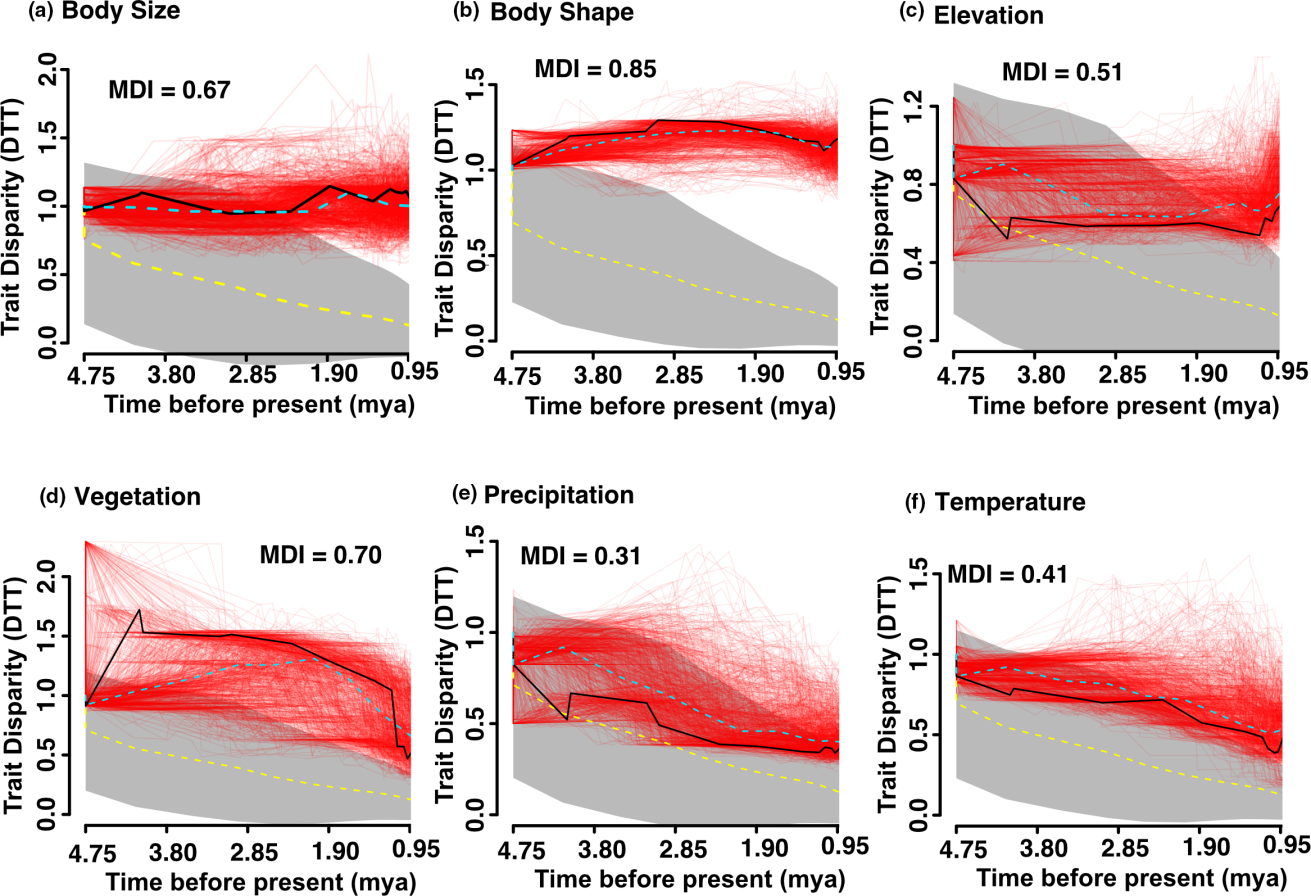
Disparity‐through‐time plots for body size (a), body shape (b), elevation (c), vegetation (d), precipitation (e), and temperature (f) in the *Liolaemus gracilis* species complex. Time (mya) is on the x‐axis, and average subclade disparity (MDI) is on the y‐axis. Gray shading indicates a null Brownian motion model of trait evolution, the yellow dotted line traces the mean of this null model. The red lines indicate 1000 random replicate trees from the posterior distribution of species trees, accounting for uncertainty in phylogenetic relationships, and timing of divergence. The mean across all these trees is shown (aqua dotted line) relative to the median species tree (black line; Figure 2). Average MDI across all 1000 posterior trees is listed in the text on the figure for each trait type

The impact of phylogenetic uncertainty varies among phenotypic and environmental traits (Figure 3), with phylogenetic uncertainty impacting the interpretation of if traits have higher relative disparity than the null (i.e., diversification in trait values) or if traits are neutrally evolving for all environmental data (Figure 3c‐f). This is especially exaggerated for vegetation (Figure 3d) which the median tree shows fits a more classic pattern seen in adaptive radiation, with increasing disparity early in the tree followed by a decline toward the present—yet posterior tree distributions indicate that broad patterns that cannot be differentiated from the null distribution of trees (gray shading). The same can be seen for precipitation (Figure 3e), yet the median tree indicates null patterns of disparity. Trends of increasing observed subclade disparity toward the present relative to the null are indicated for body size (Figure 3a) and body shape (Figure 3b). Here, phylogenetic uncertainty impacts the interpretation of the timing of such increases for body size (Figure 3a), but are striking and clear irrespective of phylogenetic uncertainty for body shape (Figure 3b), showing early and steep diversification that continues to increase toward the present.

### 3.3 Mode of environmental and phenotypic trait evolution

All individual trait datasets were shown to evolve via an OU model of trait evolution, with the exception of elevation (Table 1). For the most part, model weights were clear (with values close to or at 1) among competing models suggesting distinguishability of the model of best fit. The exception was elevation where most trees from the posterior supported a BM model (average AICw 0.65; Table 1) over the OU and EB models, but AICc differences were low (~2.57) indicating that models were not as distinguishable. Phylogenetic uncertainty seemed to have little impact on our results, given the tight confidence intervals observed on the AICw scores, parameter values (EB and OU models), and the differences in AICc scores between models tested across trees (Table 1). Body size (100% of trees), body shape (100% of trees), vegetation (95.5% of trees), precipitation (84.9%), temperature (83.4%), and elevation (100% of trees) data showed little to no evidence of significant differences between the northern and southern clades (Table 2). Again, tight confidence intervals among trees on the inferred test statistic and *p*‐value estimates indicate that phylogenetic uncertainty has little impact on results.

**TABLE 1:** Results comparing Brownian Motion (BM), Ornstein–Uhlenbeck (OU), or Early Burst (EB) models of trait evolution

Dataset	Model	Model weight	AICc	∆AICc	Parameters
Body shape	BM	0.04 (<0.01–1.0)	−704.01 ± 85.18	0–193.26	‐
	**OU**	**0.96** **(<0.01–1.0)**	**−799 ± 130.15**	**0–84.15**	**⍺ = 0.28 ± 0.80**
	EB	<0.01 (<0.01 ‐ <0.01)	−684.93 ± 83.90	22.02–211.78	β = −0.13 ± 0.15
Body size	BM	<0.01 (<0.01 ‐ <0.01)	−42.71 ± 9.16	10.13–27.03	‐
	**OU**	**1.0** **(0.99–1.0)**	**−59.83 ± 1.00**	**0–0**	**⍺ = 0.66 ± 0.09**
	EB	<0.01 (<0.01 ‐ <0.01)	−40.15 ± 9.19	12.48–29.60	β = −1.40e‐19 ± 9.04e‐18
Precipitation	BM	<0.01 (<0.01 ‐ <0.01)	366.39 ± 75.66	28.46–177.30	‐
	**OU**	**1.0** **(1.0–1.0)**	**287.73 ± 20.67**	**0–0**	**⍺ = 0.38 ± 0.76**
	EB	<0.01 (<0.01 ‐ <0.01)	369.02 ± 75.66	31.10–179.94	β = 0 ± 0
Temperature	BM	<0.01 (<0.01–0.03)	462.13 ± 80.45	6.89–166.03	‐
	**OU**	**0.99** **(0.96–1.0)**	**397.73 ± 17.27**	**0–0**	**⍺ = 0.32 ± 0.79**
	EB	<0.01 (<0.01 ‐ <0.01)	464.87 ± 80.45	9.62–168.76	β = 0 ± 0
Vegetation	BM	<0.01 (<0.01 ‐ <0.01)	290.50 ± 72.56	31.06–164.38	‐
	**OU**	**1.0** **(1.0–1.0)**	**213.46 ± 18.85**	**0–0**	**⍺ = 0.38 ± 0.76**
	EB	<0.01 (<0.01 ‐ <0.01)	293.14 ± 72.56	33.70–167.02	β = −3.19e‐12 ± 2.07e‐10
Elevation	**BM**	**0.65** **(0.64–0.64)**	**105.26 ± 9.59e‐10**	**0–0**	**‐**
	OU	0.18 (0.18–0.18)	107.83 ± 2.47e‐8	2.57–2.57	⍺ = 0.66 ± 0.09
	EB	0.18 (0.18–0.18)	107.90 ± 1.26	2.57–2.57	β = −1.72 ± 1.14

*Note*: Model fit was assessed using AICc calculated from across 1000 posterior trees. Shown are the mean and 95% confidence intervals on model weight (brackets), the mean ± standard deviation of AICc, the 95% confidence intervals on 𝚫 AICc, and the mean ± standard deviation of the ⍺ and β parameters for the OU and EB models, respectively.

**TABLE 2:** Results of tests for significant differences among northern and southern *L. gracilis* lineages, iterated across 1000 trees from the posterior distribution

Dataset	Test statistic	*p*‐value
Body shape	0.96 ± 0.03 (*0.92–0.98*)	0.95 ± 0.08 (*0.85–1.00*)
Body size^#^	0.01 ± 0.01 (*0.003–0.01*)	0.93 ± 0.02 (*0.91–0.97*)
Precipitation	0.84 ± 0.26 (*0.51–0.96*)	0.33 ± 0.41 (*0.002–0.64*)
Temperature	0.76 ± 0.34 (*0.31–0.92*)	0.27 ± 0.37 (*0.001–0.61*)
Vegetation	*0.87 ± 0.17* (*0.65–0.97*)	*0.36 ± 0.37* (*0.03* ± 0.71*)*
Elevation^#^	0.13 ± 0.09 (*0.03–0.22*)	0.72 ± 0.11 (*0.64–0.87*)

*Note*: Shown is the mean ± standard deviation test statistic (italics = 95% confidence intervals) for the multivariate phylogenetic MANOVA or univariate (^#^) phylogenetic ANOVA as appropriate. The mean ± standard deviation of the *p*‐value is also shown, with the 95% confidence intervals in italics. Analyses were undertaken using the best‐fit model of trait evolution (Table 1).

### 3.4 The evolutionary relationships between environment and phenotype

Model of evolution tests showed that all multivariate phenotype–environment correlation structures best fit the BM model (Table S5) for environmental correlations with body shape. However, the model fits for a BM model were generally marginally favored (i.e., 𝚫AICc < or = 2) but consistently favored across 1000 posterior trees (i.e., >99% of trees supported a BM model). Model weights ranged from 0.42 to 0.58 here with tight confidence intervals across trees on all estimated parameters, indicating a limited effect of phylogenetic uncertainty on the interpretation of results or favored model. For body size, the model of evolution tests showed that phenotype–environment correlation structures best fit an OU model (Table S5), with greater power to differentiate among models (i.e, 𝚫AICc <8 and > 99% of posterior trees supporting model of best fit). Again, according to the spread of estimated parameters being narrow, there seemed to be a limited effect of phylogenetic uncertainty on inferring the model of best fit for correlations with body size. Greater power to distinguish models in univariate tests, relative to multivariate analyses, may relate to the relative power needed to distinguish the best‐fit model of trait evolution.

Table 3 outlines the results from phenotype–environment correlations. Body shape is significantly (*p* < .01) correlated with vegetation PC1 which corresponds to vegetative biomass (NDVI) and seasonality in biomass. The results for body shape appear robust (i.e., low confidence limits) to phylogenetic uncertainty when estimated across 1000 posterior trees. We do not find significant, and robust, estimates of a phenotype–environment correlation between body size and any variable, although ~25% of trees favor a correlation between vegetation PC1 (mean and seasonality of NDVI), and ~42% favor a correlation with temperature (wet and cold temperature; Table 3). Confidence limits are extremely wide for the test statistic and *p*‐value estimates with most comparisons with body size yielding a few trees that supported a particular correlation. However, the same comparison may yield the directly opposing pattern in a set of trees. For example, *p*‐values range from <.001 to .95 for the correlation between elevation and body size. To test if this was due to the difference in the model, between an OU and BM, we used the OU model for body shape analyses and the BM for body size analyses (Table S5). These results showed no impact of both phylogenetic uncertainty and model choice on interpretation for multivariate analyses of correlations between body shape and environmental data. However, the univariate analyses were both susceptible to phylogenetic uncertainty, with often large confidence intervals around the test statistic and *p*‐values, but also sensitive to model choice, showing drastically more correlation between body size and environmental data.

**TABLE 3:** Results of tests for evolutionary correlations between environmental (vegetation, precipitation, temperature, and elevation) and phenotypic (body shape and body size) traits. The results for each axis of variation in vegetation, temperature, and precipitation with respect to dependent multivariate body shape and univariate body size are shown separately

Phenotypic data	Environmental data										
	Vegetation			Temperature				Precipitation			Elevation
	Av. NDVI	Tree Cover	Av. NDVI	Max Temp.	Wet Temp.	Diur. Range	Isotherm.	Ann. Prec.	Dry Prec.	Warm Prec	
	Seas. NDVI		Seas. NDVI	Warm Temp.	Cold Temp.	Ann. Range	Min. Temp.	Wet Prec.	Cold Prec		
	56.43%	37.26%	6.31%	49.03%	27.64%	18.22%	4.37%	47.68%	44.28%	7.19%	
Body shape	**0.55** **(0.54–0.55)**	0.13 (0.13–0.13)	0.07 (0.07–0.08)	0.21 (0.21–0.21)	0.18 (0.18–0.18)	0.28 (0.25–0.32)	0.25 (0.25–0.25)	0.32 (0.29–0.34)	0.09 (0.07–0.10)	0.13 (0.11–0.14)	0.13 (0.13–0.13)
*p‐value*	<0.01** **(0.009–0.009)** **100%**	0.75 (0.75–0.76) 0%	0.91 (0.91–0.91) 0.2%	0.51 (0.51–0.51) 0%	0.60 (0.60–0.60) 0%	0.14 (0.14–0.14) 0%	0.37 (0.37–0.37) 0%	0.19 (0.15–0.23) 0%	0.89 (0.84–0.92) 0%	0.76 (0.71–0.80) 0%	0.69 (0.69–0.69) 0%
Body size	7.38 (1.01–41.90)	0.79 (0.07–4.46)	1.35 (0.12–6.45)	6.61 (0.01–34.39)	8.29 (1.09–51.19)	3.16 (0.46–9.37)	0.61 (<0.01–2.58)	4.28 (0.03–22.29)	0.85 (0.13–1.83)	1.29 (0.03–7.47)	3.45 (0.004–19.84)
*p‐value*	0.09 (<0.001–0.33) 24.5%	0.55 (0.05–0.79) 2.6%	0.46 (0.02–0.74) 5.4%	0.34 (<0.001–0.92) 8.8%	0.07 (<0.001–0.31) 42.41%	0.17 (0.006–0.50) 17.7%	0.59 (0.12–0.97) 1.6%	0.24 (<0.001–0.87) 8.5%	0.43 (0.19–0.72) 0.5%	0.49 (0.01–0.86) 5.7%	0.48 (<0.001–0.95) 8.7%

*Note*: We provide the mean (large type) and 95% confidence intervals (brackets) for the test statistic, and mean and 95% confidence intervals (brackets) for slope significance *p*‐values. Parameter estimates are taken from 1000 randomly drawn trees from the posterior distribution. Trait correlations that are significant are bolded. *p*‐value ≤ .01 **. The proportions of variance described by multivariate axes (i.e., vegetation, temperature, and precipitation) are shown (large type proportions) along with the main loaded variables on those axes (see Table S4).

## 4 DISCUSSION

We focus on evolutionary patterns at shallow phylogenetic scales in the *L. gracilis* species complex with extensive taxon sampling. Our results suggest that two major lineages (Figure 2) whose body shape and size variation and possible variation in the vegetative structure of the habitats they experience have diversified recently, with phenotypic diversification increasingly prominent toward the present (Figure 3) according to an evolutionary model that evolution toward an optimum (OU; Table 1). However, these two lineages do not just represent two significantly different lineages each with its own evolutionary trajectory (Table 2). We show that body shape is the most important aspect of phenotypic variation in this clade, an often underappreciated axis of phenotypic evolution in macroevolutionary studies of animals but especially in *Liolaemus* (Figure 3). Shape evolution is evolutionarily correlated with the occupation of habitats with various vegetation structures and degrees of biomass (Table 3). Lastly, we explore important analytical caveats when undertaking evolutionary correlation analyses with respect to phylogenetic uncertainty in rapidly radiating groups and the importance of model choice between dependent multivariate and univariate tests of phenotype–environment correlation.

### 4.1 Phylogenetic relationships and timing of diversification

The *L. gracilis* species complex, which includes much of the environmental diversity and geographic diversity represented by the *Liolaemus* clade, comprises northern and southern lineages (Figures 1 & 2) that our results show diversified over the last ~4–6 million years. The evolutionary history of these two lineages differs, with an older phylogenetic history (3.3–5 million years) characterizing the northern lineage. The southern lineage, in contrast, is characterized by a relatively recent (1–2 million years) rapid diversification. Similarly, older phylogenetic divergences in northern versus younger southern lineages have been documented in several other Patagonian *Liolaemus* species complexes (Avila et al., 2006; Medina et al., 2018; Morando et al., 2003; Morando et al., 2004; Morando et al., 2007; Villamil et al., 2019). Our results concur with the phylogenetic structure and monophyly of the *L. gracilis* clade reported by Esquerré et al. (2021) using more species group representation but less complete taxon sampling within groups, albeit with shallower timing, possibly due to the inclusion of more species groups. Nevertheless, the biogeographic patterns reported in both studies are associated with dramatic glacial‐driven climatic changes and habitat shifts in southern Patagonia. Significant glaciations during the Miocene, Pliocene, and repeatedly throughout the Pleistocene and Quaternary (Ponce et al., 2011; Rabassa, 2008; Rabassa et al., 2005; Rabassa et al., 2011) left many northern regions ice‐free which likely served as refugia during periods of glaciation considering the age of *L. gracilis* complex clades from our analysis (Figure 2). However, southern Patagonian habitats were either under permafrost or shifted off the current coastline by up to four degrees during glaciation (Ponce et al., 2011; Rabassa, 2008; Rabassa et al., 2005; Rabassa et al., 2011), which could have resulted in the shallower phylogenetic history of the southern *L. gracilis* clade. Nevertheless, deeper phylogenetic histories were found in the *Liolaemus lineomaculatus* (Breitman et al., 2012) and *Liolaemus elongatus* (Medina et al., 2018) groups suggest that this shallow phylogenetic history may not be entirely due to a lack of refugia in the south. Shallow histories may simply be a consequence of a natural southern expansion of the southern *L. gracilis* clade or competitive exclusion by other diverse *Liolaemus* clades, further investigation is warranted here.

### 4.2 Patterns of ecological and phenotypic diversification

Despite long being recognized as a source of error in comparative analyses (Huelsenbeck et al., 2000), methods accounting for phylogenetic uncertainty have only recently become available. Phylogenetic uncertainty within *Liolaemus* is not entirely unexpected, given the well‐documented cases of interspecific introgression observed among species (e.g., Esquerré et al., 2021; Olave et al., 2011; Olave et al., 2018), the rapidity with which species have accumulated, and the small number of loci used for phylogenetic studies for most species within *Liolaemus* species groups (Heled & Drummond, 2010). We found that phylogenetic uncertainty impacts the interpretation of patterns of observed subclade disparity, specifically, interpretations based on the median tree often misrepresent patterns exhibited across the posterior distribution of trees (Figure 3). This result has also been observed in other studies that consider phylogenetic uncertainty in analyses of disparity‐through‐time (Colombo et al., 2015; Edwards et al., 2015; Foth et al., 2017; Pearman et al., 2014). For all environmental datasets, phylogenetic uncertainty results in an inability to determine if traits are diversifying or evolving according to the null model. An important caveat here is of course species may have not remained static in their environmental preferences, and these analyses implicitly assume that they have. Nevertheless, the structural vegetation environment species experience may have either evolved according to a null model or rapidly diversified early in the history of the *L. gracilis* clade, with more recent declines in the diversity of environments toward the present. Diversification in environmental vegetation structure has been observed in other lizards (Edwards et al., 2015), but such studies are rare in other animal groups.

For body size and shape, the impacts of phylogenetic uncertainty do not actively impede the interpretation of patterns of diversification, where both shows increasing diversification toward the present (Figure 3). Our results indicate that body shape is the most labile aspect of phenotypic diversification in the *L. gracilis* clade, much more so than body size. There are many studies that suggest body shape diversification has been important for multiple vertebrate groups (Allen et al., 2013; Bonett & Blair, 2017; Friedman et al., 2019, 2020; Goodman et al., 2009; Gray et al., 2019; Vanhooydonck & Damme, 1999). Therefore, contrary to numerous studies and higher phylogenetic scales (i.e., Espinoza et al., 2004; Harmon et al., 2003), our study at a shallower phylogenetic scale and with detailed sampling, does not support the hypothesis that body size is a major axis of variation in *Liolaemus*. This result concurs with more recent studies with more extensive taxon sampling, showing that body size evolution does not correspond to diversification in *Liolaemus* (Olave et al., 2020). Nevertheless, there are inconsistent results across vertebrates and invertebrates in terms of patterns of body size diversification (Cooper & Purvis, 2010; Itescu et al., 2018; Laurin, 2004; Pallarés et al., 2019; Rainford et al., 2016). The finding that body shape evolution may be more extensive and possibly independent of body size evolution has been found in fishes (Colombo et al., 2015; Friedman et al., 2019), but see (Hendry et al., 2006), and lizards (Goodman et al., 2009). Although such studies that compare body shape diversification directly to body size diversification are rare, and our results imply these may warrant further study.

### 4.3 Mode of phenotypic trait evolution

We show that the evolution of environmental and phenotypic traits, individually, clearly follow an OU model (Table 1) and we find no evidence that the northern and southern lineages significantly differ in the environmental and phenotypic axes tested (Table 2). Our results indicate that the traits we tested seem to be evolving according to adaptive processes, which is consistent with analyses of body size data across *Liolaemus* (Olave et al., 2020). This study is the first to show that an OU process fits the evolution of a variety of environmental and phenotypic features, which has not been tested broadly across *Liolaemus*. This has largely been due to shape either not being considered variable (Jaksić et al., 1980) or irrelevant to adaptive variation due to a lack of phenotype–environment correlations (Schulte et al., 2004). This warrants further investigation across liolaemids. OU models generally model stabilizing selection toward an adaptive optimum (Beaulieu et al., 2012), but nevertheless have their limitations, including high Type‐I error rates with limited sample sizes and difficulty interpreting parameter values (Cooper et al., 2016). Therefore, we must view our results within these caveats.

### 4.4 The evolutionary relationships between environment and phenotype

Our results show that body size has limited or no evolutionary relationship with any environmental variable tested in *L. gracilis* species (Table 3; but see discussion below of potential methodological issues). This contrasts with studies in other *Liolaemus* species where correlated evolution between body size and temperature have been reported (Azócar et al., 2015; Espinoza et al., 2004), but see (Jaksić et al., 1980; Pincheira‐Donoso et al., 2007). Such variation in evolutionary relationships between body size and environmental variables among ectotherm clades is common because habitat preferences may vary and evolutionary lability in size is not necessarily consistent across clades (Pallarés et al., 2019). Our results show a clear and robust correlation between body shape and vegetation, specifically biomass average and seasonality (Table 3). Yet, studies across *Liolaemus* have failed to find consistent evidence that the evolution of environmental and body shape traits are related (Jaksić et al., 1980; Pincheira‐Donoso et al., 2009; Schulte et al., 2004; Tulli et al., 2012). Body shape variation is particularly important for functional locomotory performance in lizards (Bergmann & Irschick, 2010; Garland & Losos, 1990; Melville & Swain, 2000) that are also important in other animals (Irschick et al., 2008). High biomass and low seasonality versus low biomass with high seasonality typify the Argentinian humid northwest Andes, and the southern Patagonian cold and arid steppe environments, respectively (Fabricante et al., 2009; León et al., 1998). Therefore, such differences in habitat structure likely impact escape behavior, which has been associated with body shape in *Liolaemus* (Schulte et al., 2004), and selection of body shape in these vastly different environments.

### 4.5 Phylogenetic uncertainty and model choice in studies of evolutionary correlation

One important limitation of our study appears to be the robustness of the results of our phenotype–environment correlations to the impact of phylogenetic uncertainty and the differences between tests involving multivariate versus univariate dependent variables, impacting our ability to interpret if there are phenotypic–environment correlations with body size. Multivariate and univariate dependent trait analyses are necessarily conducted using different approaches, as there are currently no methods to the best of our knowledge that allow direct comparisons. Nevertheless, we show that analyses of phenotype–environment correlations with univariate dependent traits are more susceptible to both phylogenetic uncertainty (wide confidence intervals on test statistics, *p*‐values, and parameter estimates) and model misspecification than those with multivariate dependent data. For example, with an OU model, there are no significant phenotype–environment associations with body size, but with a BM model there are significant relationships between body size and vegetation biomass and temperature (Table S6). This contrasts with studies indicating that the univariate phylogenetic regression is robust to tree misspecification, but can be impacted by extreme variation in topological and branch length reconstruction (Stone, 2011). Likely important here given hybridization in *Liolaemus* (Esquerré et al., 2021; Olave et al., 2018). We are not aware of any such explorations of the robustness of multivariate regression approaches considering tree misspecification, nevertheless, our results suggest they may be more robust to phylogenetic uncertainty.

Model misspecification has been noted with increasing trait dimensions in multivariate analyses (Adams & Collyer, 2018, 2019), and it may be that our dataset here is small enough to avoid such artifacts. Nevertheless, model misspecification is clearly noted as impacting the interpretation of univariate dependent trait phylogenetic regression analyses and an OU model is often chosen for such datasets despite not being able to adequately explain the correlation structure (Pennell et al., 2015). Early burst models are rare in comparative data (Harmon et al., 2010), therefore, it may be that a BM model does explain the correlation structure of phenotype–environment correlations here, and that body size does correlate with environmental features. Regardless, our results indicate that further explorations of the relative robustness of multivariate and univariate phylogenetic regression approach with respect to model and tree misspecification may be warranted.

## 5 CONCLUSIONS

Body shape is an important predictor of performance, escape behavior, and an animals function in its environment. We show that body shape has diversified more than body size in the *L. gracilis* clade and that the evolution of shape in this group is correlated with vegetation, likely reflecting a relationship with locomotory performance and escape tactics in different structural environments. While our results for relationships between environmental variables and body size are less clear, body size has diversified toward the present in this clade. It will be important for future studies to consider the relative importance of size versus shape in adaptive radiations. Not incorporating phylogenetic uncertainty has likely impacted the interpretation of adaptive evolution in *Liolaemus* but also many other adaptive radiations. It is critical that in analyses of adaptive radiations and rapidly radiating clades that the impact of phylogenetic uncertainty be included, particularly because topological and branch length variation is highest in these clades. Interestingly, fundamental differences between methods testing multivariate versus univariate dependent variables may fundamentally differ in their sensitivity to model and tree misspecification and should be explored through simulation studies, ideally also with a unified statistical approach that can incorporate both these data types.

## AUTHOR CONTRIBUTIONS

**Danielle L. Edwards:** Conceptualization (lead); data curation (equal); formal analysis (lead); methodology (lead); writing – original draft (lead); writing – review and editing (lead). **Luciano J. Avila:** Conceptualization (supporting); data curation (equal); funding acquisition (equal); investigation (supporting); project administration (equal); supervision (equal); writing – review and editing (supporting). **Lorena Martinez:** Data curation (equal); investigation (equal). **Jack W. Sites Jr.:** Conceptualization (supporting); funding acquisition (equal); project administration (supporting); resources (equal); writing – review and editing (supporting). **Mariana Morando:** Conceptualization (supporting); data curation (supporting); funding acquisition (equal); investigation (equal); project administration (equal); supervision (lead); writing – review and editing (supporting).

## CONFLICT OF INTEREST

The authors declare no conflict of interest.

## Supporting information


Appendix S1

## Data Availability

The data that support the findings of this study are available in Dryad (https://doi.org/10.6071/M36X1M). Some data (Genbank numbers, morphological, and spatial data) are also in the Supplementary Material, which can also be found in Dryad.
