# Supplementary material for: Environmental correlates of phenotypic evolution in ecologically diverse Liolaemus lizards
Source: Ecol Evol. 2022 Jun 16;12(6):e9009. doi: 10.1002/ece3.9009 (PMC9201750; doi:10.1002/ece3.9009)
Supplement: Supplementary file 1 — Appendix S1 [file ECE3-12-e9009-s001.docx]

**Table S1:**  List of specimens sequenced and measured for morphological analyses within the current study. Genbank numbers are provided for sequences used in this study.

| Clade | Number | Latitude | Longitude | 4th Toe Length | 3rd Finger Length | Snout-vent Length | Axillo-groin Length | Arm Length | Tibia Length | Foot Length | Pelvic Width | 12S | CytB | LPA11E | LPB4G | LPB9C | LPB11E |
| --- | --- | --- | --- | --- | --- | --- | --- | --- | --- | --- | --- | --- | --- | --- | --- | --- | --- |
| Lb clade1 | LJAMM 3222 | -42.396111 | -67.333333 | 22 | 17 | 57.3 | 26.6 | 16.09 | 10.65 | 16.37 | 7.34 |  |  |  |  |  |  |
| Lb clade1 | LJAMM 6058 | N/A | N/A | N/A | N/A | N/A | N/A | N/A | N/A | N/A | N/A | ON507850 | ON507834 | ON507786 | ON507810 | ON507886 | ON507911 |
| Lb clade1 | LJAMM 6215 | -41.865833 | -68.068333 | 22 | 16 | 58.7 | 26.6 | 15.71 | 10.29 | 17.09 | 7.79 |  |  |  |  |  |  |
| Lb clade1 | LJAMM 6291 | -42.516667 | -68.283333 | 24 | 17 | 59.6 | 28.8 | 16.4 | 11.33 | 18.04 | 7.85 |  |  |  |  |  |  |
| Lb clade1 | LJAMM 6302 | -42.516667 | -68.283333 | 24 | 17 | 57.8 | 27.2 | 15.21 | 10.44 | 16.6 | 7.92 |  |  |  |  |  |  |
| Lb clade1 | LJAMM 6941 | N/A | N/A | N/A | N/A | N/A | N/A | N/A | N/A | N/A | N/A | ON507851 | ON507835 | ON507790 | ON507814 | ON507890 | ON507916 |
| Lb clade 10 | LJAMM 5340 | -37.971944 | -70.643889 | 23 | 16 | 49 | 24.32 | 13.89 | 8.87 | 14.77 | 6.03 |  |  |  |  |  |  |
| Lb clade 10 | LJAMM 5343 | -37.971944 | -70.643889 | 24 | 18 | 58 | 26.14 | 15.37 | 10.49 | 16.17 | 7.1 |  |  |  |  |  |  |
| Lb clade 10 | LJAMM 5344 | -37.971944 | -70.643889 | 25 | 17 | 52.5 | 25.41 | 13.69 | 9.49 | 14.19 | 6.38 |  |  |  |  |  |  |
| Lb clade 10 | LJAMM 6469 | -37.251389 | -70.620278 | 22 | 15 | 54 | 23.81 | 14.46 | 9.49 | 15.75 | 6.69 | ON507870 | ON507844 | ON507789 | ON507813 | ON507889 |  |
| Lb clade 11 | LJAMM 5319 | N/A | N/A | N/A | N/A | N/A | N/A | N/A | N/A | N/A | N/A |  | DQ989776 | ON507785 | ON507809 | ON507885 |  |
| Lb clade 11 | LJAMM 5320 | -37.21 | -70.74 | 22 | 16 | 47.4 | 21.6 | 12.67 | 8.2 | 14.22 | 5.98 |  |  |  |  |  |  |
| Lb clade 11 | LJAMM 5321 | -37.21 | -70.74 | 24 | 16 | 58.1 | 28.11 | 15.17 | 10.09 | 15.48 | 7.39 |  |  |  |  |  |  |
| Lb clade 12 | LJAMM 5363 | -38.832222 | -70.032778 | 22 | 16 | 53.4 | 25.63 | 14.29 | 9.87 | 14.53 | 7.11 |  |  |  |  |  |  |
| Lb clade 12 | LJAMM 5364 | -38.832222 | -70.032778 | 24 | 16 | 50.5 | 23.29 | 13.3 | 9.07 | 14.16 | 7.05 |  |  |  |  |  |  |
| Lb clade 12 | LJAMM 5395 | -39.043611 | -70.222778 | 24 | 16 | 56.3 | 28.42 | 15.57 | 10.5 | 15.62 | 7.78 |  |  |  |  |  |  |
| Lb clade 12 | LJAMM 10422 | N/A | N/A | N/A | N/A | N/A | N/A | N/A | N/A | N/A | N/A | JN410389 | JN410526 | JN410407 | JN410445 | ON507879 |  |
| Lb clade 14 | LJAMM 3773 | -44.615 | -69.1425 | 21 | 18 | 48.4 | 21.05 | 14.11 | 9.23 | 15.43 | 6.2 |  |  |  |  |  |  |
| Lb clade 14 | LJAMM 9166 | N/A | N/A | N/A | N/A | N/A | N/A | N/A | N/A | N/A | N/A |  | ON507826 | ON507798 |  | ON507898 |  |
| Lb clade 14 | LJAMM 9167 | -44.529167 | -69.180556 | 24 | 17 | 54.6 | 24.09 | 15.36 | 10.4 | 16.58 | 7.34 | ON507863 | ON507827 | ON507799 | ON507822 | ON507899 | ON507924 |
| Lb clade 15 | LJAMM 6281 | -40.132778 | -68.939722 | 20 | 15 | 49.3 | 19.7 | 13.56 | 8.88 | 13.92 | 7.36 |  |  |  |  |  |  |
| Lb clade 15 | LJAMM 6284 | -40.132778 | -68.939722 | 19 | 14 | 52.7 | 24.4 | 13.97 | 9.26 | 14.14 | 7.04 |  |  |  |  |  |  |
| Lb clade 15 | LJAMM 6286 | -40.132778 | -68.939722 | 21 | 16 | 46.1 | 20.8 | 12.66 | 7.9 | 13.97 | 6.42 |  |  |  |  |  |  |
| Lb clade 15 | LJAMM 6287 | N/A | N/A | N/A | N/A | N/A | N/A | N/A | N/A | N/A | N/A | ON507861 | ON507838 | ON507788 | ON507812 | ON507888 | ON507914 |
| Lb clade 16 | LJAMM 6847 | -40.2925 | -68.923611 | 24 | 16 | 52.5 | 24.15 | 14.38 | 9.48 | 16.17 | 6.6 | JN410370/ON507862 | JN410524 | JN410433 | JN410471 | JN410506 | ON507915 |
| Lb clade 16 | LJAMM 6864 | -40.286111 | -68.459722 | 25 | 16 | 59.7 | 26.3 | 15.57 | 10.82 | 17.01 | 7.78 |  |  |  |  |  |  |
| Lb clade 16 | LJAMM 6865 | -40.286111 | -68.459722 | 23 | 16 | 56.1 | 24.98 | 15.38 | 10.08 | 16.56 | 7.49 |  |  |  |  |  |  |
| Lb clade 16 | LJAMM 6866 | -40.286111 | -68.459722 | 22 | 15 | 59.8 | 25.14 | 15.74 | 10.9 | 15.46 | 7.7 |  |  |  |  |  |  |
| Lb clade 16 | LJAMM 6867 | -40.286111 | -68.459722 | 22 | 15 | 52.9 | 23.09 | 14.59 | 9.63 | 16.3 | 6.7 |  |  |  |  |  |  |
| Lcyaneinotatus | LJAMM 10375 | N/A | N/A | N/A | N/A | N/A | N/A | N/A | N/A | N/A | N/A | ON507874 | ON507846 | ON507779 | ON507802 | ON507877 | ON507903 |
| Lcyaneinotatus | LJAMM 10383 | N/A | N/A | N/A | N/A | N/A | N/A | N/A | N/A | N/A | N/A | ON507875 | ON507845 | ON507780 | ON507803 | ON507878 | ON507904 |
| Lcyaneinotatus | LJAMM 10384 | -37.690833 | -68.803056 | 22 | 15 | 57.71 | 25.14 | 20.92 | 10.31 | 15.05 | 6.78 |  |  |  |  |  |  |
| Lcyaneinotatus | LJAMM 10385 | -37.690833 | -68.803056 | 24 | 16 | 51.26 | 24.05 | 14.82 | 9.62 | 16.94 | 6.76 |  |  |  |  |  |  |
| Lcyaneinotatus | LJAMM 10386 | -37.690833 | -68.803056 | 22 | 16 | 54.47 | 26.36 | 14.19 | 10.49 | 15 | 6.79 |  |  |  |  |  |  |
| Lcyaneinotatus | LJAMM 10388 | N/A | N/A | N/A | N/A | N/A | N/A | N/A | N/A | N/A | N/A | JN410393 | JN410531 | JN410406 | JN410444 | JN410482 | ON507905 |
| Lcyaneinotatus | LJAMM 10389 | -37.723056 | -68.888333 | 24 | 18 | 59 | 25.47 | 15.93 | 10.77 | 17.41 | 7.72 |  |  |  |  |  |  |
| Lcyaneinotatus | LJAMM 10551 | -37.725833 | -68.926111 | 24 | 19 | 53.05 | 25.01 | 15.16 | 10.01 | 16.16 | 6.69 |  |  |  |  |  |  |
| Lb clade 17 | LJAMM 8710 | N/A | N/A | N/A | N/A | N/A | N/A | N/A | N/A | N/A | N/A | ON507872 | ON507848 | ON507795 | ON507819 | ON507895 | ON507920 |
| Lb clade 17 | LJAMM 8902 | N/A | N/A | N/A | N/A | N/A | N/A | N/A | N/A | N/A | N/A | ON507873 | ON507849 | ON507796 | ON507820 | ON507896 | ON507921 |
| Lb clade 19 | LJAMM 10445 | N/A | N/A | N/A | N/A | N/A | N/A | N/A | N/A | N/A | N/A | ON507871 | ON507847 |  | ON507804 | ON507880 |  |
| Lb clade 2 | LJAMM 4544 | N/A | N/A | N/A | N/A | N/A | N/A | N/A | N/A | N/A | N/A | ON507852 | ON507836 | ON507784\ | ON507808 | ON507884 | ON507910 |
| Lb clade 2 | LJAMM 8960 | -43.468333 | -68.6575 | 19 | 17 | 55.8 | 23.4 | 15.8 | 11.01 | 17.66 | 7.04 |  |  |  |  |  |  |
| Lb clade 2 | LJAMM 8974 | -43.080556 | -70.355556 | 22 | 15 | 53.2 | 21.57 | 14.29 | 9.98 | 14.32 | 6.65 |  |  |  |  |  |  |
| Lb clade 2 | LJAMM 8984 | N/A | N/A | N/A | N/A | N/A | N/A | N/A | N/A | N/A | N/A | ON507853 | ON507837 | ON507797 | ON507821 | ON507897 | ON507922 |
| Lb clade 2 | LJAMM 8986 | -42.664167 | -70.372222 | 22 | 16 | 51.2 | 23.8 | 14.27 | 10.03 | 15.82 | 6.79 |  |  |  |  |  |  |
| Lb clade 2 | LJAMM 8988 | N/A | N/A | N/A | N/A | N/A | N/A | N/A | N/A | N/A | N/A | JN4103838 | JN410550 | JN410440 | JN410478 | JN410513 | ON507923 |
| Lb clade 2 | LJAMM 9048 | -43.595833 | -70.164722 | 24 | 16 | 59 | 25.74 | 16.22 | 10.91 | 18.07 | 8.17 |  |  |  |  |  |  |
| L. vhagar sp. A | LJAMM 5538 | -40.325278 | -69.438333 | 21 | 16 | 51.1 | 24.5 | 14.36 | 9.97 | 15.37 | 6.55 |  |  |  |  |  |  |
| L. vhagar sp. A | LJAMM 5540 | -40.325278 | -69.438333 | 24 | 16 | 56.4 | 28.1 | 14.5 | 9.95 | 15.86 | 7.79 |  |  |  |  |  |  |
| L.vhagar sp. A | LJAMM 6278 | -40.295556 | -68.969167 | 24 | 15 | 53 | 22.9 | 14.37 | 9.35 | 15.86 | 6.77 | JN410372 | JN410545 | JN410429 | JN410467 | JN410502 | ON507913 |
| L.vhagar sp. A | LJAMM 6279 | -40.132778 | -68.939722 | 24 | 17 | 51.4 | 22.2 | 15.02 | 10 | 16.95 | 6.92 |  |  |  |  |  |  |
| L. vhagar sp. A | LJAMM 8685 | N/A | N/A | N/A | N/A | N/A | N/A | N/A | N/A | N/A | N/A | ON507857 | ON507841 | ON507794 | ON507818 | ON507894 |  |
| Lb clade 3b | LJAMM 8053 | -39.084722 | -70.373056 | 24 | 17 | 54.4 | 24.7 | 14.82 | 9.11 | 15.95 | 6.82 |  |  |  |  |  |  |
| Lb clade 3b | LJAMM 8091 | N/A | N/A | N/A | N/A | N/A | N/A | N/A | N/A | N/A | N/A | ON507858 | ON507840 | ON507792 | ON507816 | ON507892 | ON507918 |
| L. vhagar sp. C | LJAMM 6272 | -39.410056 | -68.457417 | 23 | 18 | 53.6 | 25.2 | 15.12 | 10.15 | 15.59 | 6.98 | ON507856 | ON507839 |  |  |  |  |
| L. balerion sp. A | LJAMM 3518 | -41.703333 | -70.484167 | 23 | 16 | 57.2 | 25 | 15.83 | 10.96 | 17 | 7.58 |  |  |  |  |  |  |
| L. balerion sp. A | LJAMM 3529 | -41.756667 | -70.530278 | 20 | 16 | 57.5 | 26.14 | 16.13 | 10.65 | 16.54 | 7.05 |  |  |  |  |  |  |
| L. balerion sp. A | LJAMM 5419 | -40.878611 | -70.574444 | 21 | 16 | 51.3 | 22.92 | 14.63 | 10.04 | 17.36 | 6.64 |  |  |  |  |  |  |
| L. balerion sp. A | LJAMM 5649 | -40.875278 | -70.020556 | 21 | 16 | 61.8 | 30.83 | 16.14 | 11.12 | 15.88 | 7.92 |  |  |  |  |  |  |
| L. balerion sp. A | LJAMM 6277 | -42.004167 | -70.669722 | 23 | 18 | 57.4 | 28.94 | 14.92 | 10.86 | 16.64 | 7 | ON507855 | ON507842 | ON507787 | ON507811 | ON507887 | ON507912 |
| L. balerion sp. A | LJAMM 3486 | N/A | N/A | N/A | N/A | N/A | N/A | N/A | N/A | N/A | N/A | JN410396/ON507854 | JN410528 | JN410423 | JN410460 | JN410495 | ON507909 |
| Lb clade 5 | LJAMM 933 | -39.541389 | -70.955833 | 24 | 17 | 49.4 | 22.77 | 13.04 | 9.76 | 14.34 | 6.46 | ON507859 | DQ989784 | ON507800 | ON507823 | ON507900 |  |
| Lb clade 6 | LJAMM 1645 | N/A | N/A | N/A | N/A | N/A | N/A | N/A | N/A | N/A | N/A | JN410381 | JN410525/DQ989788 | JN410420 | JN410457 | JN410492 |  |
| L.balerion sp. b | LJAMM 2339 | -41.541667 | -69.859167 | 22 | 16 | 57.3 | 25.7 | 15.71 | 10.69 | 16.68 | 7.78 |  |  |  |  |  |  |
| L.balerion sp. b | LJAMM 3587 | -41.365278 | -69.808333 | 23 | 17 | 59.6 | 25.95 | 16.1 | 11.13 | 16.72 | 7.24 | ON507860 | ON507825 | ON507783 | ON507807 | ON507883 |  |
| Lb clade 8a | LJAMM 1388 | -36.6325 | -69.8375 | 23 | 18 | 55.5 | 24.26 | 15.48 | 10.28 | 16.43 | 7.63 |  |  |  |  |  |  |
| Lb clade 8a | LJAMM 2558 | -36.638889 | -69.831667 | 24 | 17 | 53.7 | 24.37 | 16.41 | 10.22 | 17.33 | 7.34 |  |  |  |  |  |  |
| Lb clade 8a | LJAMM 7774 | -36.43 | -69.802778 | 24 | 16 | 50.1 | 22.51 | 14.68 | 9.71 | 15.49 | 6.71 | JN410369 | JN410534 | JN410435 | JN410473 | JN410508 | ON507917 |
| Lb clade 8a | LJAMM 7776 | -36.43 | -69.802778 | 23 | 17 | 51.7 | 21.67 | 14.56 | 10.24 | 15.92 | 7.11 |  |  |  |  |  |  |
| L. meraxes sp. B | LJAMM 7918 | -35.796667 | -70.106944 | 24 | 16 | 51.7 | 23.82 | 15.03 | 9.71 | 16.43 | 7 |  |  |  |  |  |  |
| L. meraxes sp. B | LJAMM 7920 | -35.796667 | -70.106944 | 24 | 16 | 51.1 | 21.38 | 14.24 | 9.7 | 15.65 | 7.22 |  |  |  |  |  |  |
| L. meraxes sp. B | LJAMM 7921 | N/A | N/A | N/A | N/A | N/A | N/A | N/A | N/A | N/A | N/A | ON507869 | ON507843 | ON507791 | ON507815 | ON507891 |  |
| L. meraxes sp. B | LJAMM 8002 | -35.518056 | -69.825833 | 23 | 15 | 50.6 | 21.79 | 14.51 | 8.98 | 16.63 | 6.51 |  |  |  |  |  |  |
| Lb clade 9 | LJAMM 5258 | -36.659722 | -70.584722 | 22 | 15 | 48.9 | 22.16 | 13.03 | 9.15 | 14.28 | 6.59 |  |  |  |  |  |  |
| Lb clade 9 | LJAMM 5274 | -36.659722 | -70.584722 | 22 | 16 | 51.72 | 24.9 | 12.42 | 8.84 | 13.97 | 6.69 |  |  |  |  |  |  |
| Lb clade 9 | LJAMM 5275 | -36.659722 | -70.584722 | 22 | 15 | 52.5 | 24.12 | 13.84 | 8.98 | 14.97 | 6.87 |  |  |  |  |  |  |
| Lb clade 9 | LJAMM 5279 | -36.9325 | -70.917778 | 22 | 16 | 44.76 | 20.86 | 12.74 | 8.54 | 13.67 | 6.8 |  |  |  |  |  |  |
| Lb clade 9 | LJAMM 5290 | -36.9325 | -70.917778 | 23 | 16 | 46 | 20.79 | 11.96 | 7.65 | 13.62 | 5.77 |  |  |  |  |  |  |
| Lb clade 9 | LJAMM 6436 | -36.659722 | -70.584444 | 22 | 16 | 54.1 | 24.93 | 14.61 | 9.52 | 14.86 | 6.81 | JN410387 | JN410530 | JN410431 | JN410469 | JN410504 |  |
| Lb clade 9 | LJAMM 6437 | -36.659722 | -70.584444 | 21 | 15 | 46.6 | 21.3 | 12.89 | 8.72 | 13.85 | 6.31 |  |  |  |  |  |  |
| Lbibronii | LJAMM 3227 | N/A | N/A | N/A | N/A | N/A | N/A | N/A | N/A | N/A | N/A | ON507864 | ON507831 | ON507781 | ON507805 | ON507881 | ON507906 |
| Lbibronii | LJAMM 3249 | -41.581111 | -66.5075 | 22 | 17 | 52.8 | 22.1 | 15.44 | 9.79 | 16.06 | 7.01 |  |  |  |  |  |  |
| Lbibronii | LJAMM 3353 | N/A | N/A | N/A | N/A | N/A | N/A | N/A | N/A | N/A | N/A | ON507868 | ON507828 | ON507782 | ON507806 | ON507882 | ON507907 |
| Lbibronii | LJAMM 3367 | -40.796667 | -68.036667 | 22 | 16 | 58 | 27.71 | 15.51 | 10.46 | 15.52 | 7.89 | JN410382 | ON507829 | JN410422 | JN410459 | JN410494 | ON507908 |
| Lbibronii | LJAMM 3747 | -44.259444 | -68.259444 | 24 | 16 | 57.3 | 25.64 | 14.86 | 10.16 | 16.15 | 6.94 |  |  |  |  |  |  |
| Lbibronii | LJAMM 3751 | -44.259444 | -68.259444 | 24 | 17 | 49.57 | 23.17 | 14.36 | 9.5 | 16.38 | 6.59 |  |  |  |  |  |  |
| Lbibronii | LJAMM 7475 | -47.715 | -65.839167 | 22 | 15 | 46.13 | 21.4 | 12.64 | 8.35 | 14 | 5.95 |  |  |  |  |  |  |
| Lbibronii | LJAMM 8210 | N/A | N/A | N/A | N/A | N/A | N/A | N/A | N/A | N/A | N/A | ON507865 | ON507832 | ON507793 | ON507817 | ON507893 | ON507919 |
| Lbibronii | LJAMM 9888 | -47.715 | -65.839167 | 26 | 19 | 52.64 | 24.28 | 14.24 | 10.41 | 14.84 | 7.24 |  |  |  |  |  |  |
| Lbibronii | LJAMM 9889 | -47.715 | -65.839167 | 21 | 18 | 50.9 | 21.57 | 14.58 | 9.91 | 14.65 | 7.28 |  |  |  |  |  |  |
| Lbibronii | LJAMM 9890 | -47.715 | -65.839167 | 22 | 15 | 55.6 | 26.04 | 15.94 | 10.66 | 16.46 | 7.45 |  |  |  |  |  |  |
| Lbibronii | LJAMM 9897 | N/A | N/A | N/A | N/A | N/A | N/A | N/A | N/A | N/A | N/A | ON507866 | ON507833 | ON507801 | N507824 | ON507901 | ON507925 |
| Lbibronii | LJAMM 9925 | -46.9675 | -68.416111 | 24 | 19 | 53.33 | 23.28 | 12.29 | 10.51 | 14.75 | 7.29 |  |  |  |  |  |  |
| Lbibronii | LJAMM 10307 | N/A | N/A | N/A | N/A | N/A | N/A | N/A | N/A | N/A | N/A | ON507867 | ON507830 | ON507778 |  | ON507876 | ON507902 |
| Lgracilis | LJAMM 147 | -38.116667 | -67.1 | 24 | 18 | 48.04 | 22.66 | 12.75 | 9.73 | 14.97 | 6.53 |  |  |  |  |  |  |
| Lgracilis | LJAMM 150 | -38.116667 | -67.1 | 25 | 16 | 52.67 | 22.85 | 13 | 9.7 | 14.51 | 6.08 |  |  |  |  |  |  |
| Lgracilis | LJAMM 152 | -38.116667 | -67.1 | 23 | 17 | 51.76 | 23.09 | 13.76 | 9.69 | 14.8 | 6.46 |  |  |  |  |  |  |
| Lgracilis | LJAMM 2572 | -40.840556 | -65.117778 | 22 | 16 | 48.47 | 21.31 | 12.81 | 9.08 | 13.96 | 5.56 |  |  |  |  |  |  |
| Lgracilis | LJAMM 3330 | -42.615 | -64.173889 | 24 | 18 | 48.16 | 20.66 | 12.73 | 8.07 | 13.49 | 5.75 |  |  |  |  |  |  |
| Lgracilis | LJAMM 3332 | -42.615 | -64.173889 | 23 | 17 | 48.99 | 21.6 | 12.25 | 8.79 | 13.09 | 5.47 |  |  |  |  |  |  |
| Lgracilis | LJAMM 3333 | -42.615 | -64.173889 | 23 | 16 | 48.7 | 22.78 | 12.13 | 8.65 | 12.98 | 5.23 |  |  |  |  |  |  |
| Lgracilis | LJAMM 3341 | -42.615 | -64.173889 | 21 | 15 | 48.03 | 22.61 | 12.31 | 8.03 | 14.01 | 5.34 |  |  |  |  |  |  |
| Lgracilis | LJAMM 4542 | N/A | N/A | N/A | N/A | N/A | N/A | N/A | N/A | N/A | N/A | JN410397 | JN410538 | JN410426 | JN410464 | JN410499 |  |
| Lgracilis | LJAMM 5699 | -38.184694 | -69.022917 | 24 | 15 | 49.04 | 22.75 | 13.48 | 9.33 | 15.83 | 6.1 |  |  |  |  |  |  |
| Lgracilis | LJAMM 5701 | -38.184694 | -69.022917 | 24 | 17 | 45.09 | 19.35 | 12.65 | 9.18 | 15.4 | 6.19 |  |  |  |  |  |  |
| Lgracilis | LJAMM 5711 | -38.184694 | -69.022917 | 23 | 16 | 48.68 | 22.49 | 14.12 | 9.79 | 13.95 | 5.96 |  |  |  |  |  |  |
| Lgracilis | LJAMM 5712 | -38.184694 | -69.022917 | 23 | 17 | 51.14 | 23.48 | 13.36 | 9.85 | 14.08 | 6.94 |  |  |  |  |  |  |
| Lgracilis | LJAMM 7114 | -39.666111 | -68.410139 | 23 | 16 | 52.8 | 22.65 | 13.4 | 9.62 | 15.62 | 6.19 |  |  |  |  |  |  |
| Lgracilis | LJAMM 7695 | N/A | N/A | N/A | N/A | N/A | N/A | N/A | N/A | N/A | N/A | JN410385 | JN410546 | JN410434 | JN410472 | JN410507 |  |
| Lgracilis | LJAMM 8907 | N/A | N/A | N/A | N/A | N/A | N/A | N/A | N/A | N/A | N/A | JN410400 | JN410552 | JN410439 | JN410477 | JN410512 |  |
| Lgracilis | LJAMM 10348 | N/A | N/A | N/A | N/A | N/A | N/A | N/A | N/A | N/A | N/A | JN410402 | JN410523 | JN410405 | JN410443 | JN410481 |  |
| Lramirezae | LJAMM 12648 | -27.366361 | -66.373611 | 24 | 16 | 52.1 | 22.88 | 13.59 | 9.1 | 15.8 | 5.8 |  |  |  |  |  |  |
| Lramirezae | LJAMM 12649 | -27.366361 | -66.373611 | 19 | 15 | 49.79 | 18.61 | 11.74 | 8.36 | 14.48 | 5.57 |  |  |  |  |  |  |
| Lramirezae | LJAMM 12695 | -26.755444 | -65.818167 | 25 | 15 | 56.32 | 28.22 | 13.91 | 9.48 | 14.32 | 8.02 |  |  |  |  |  |  |
| Lramirezae | LJAMM 12750 | -25.237361 | -65.900722 | 21 | 18 | 53.5 | 26.52 | 13.41 | 9.41 | 14.77 | 7.19 |  |  |  |  |  |  |
| Lramirezae | LJAMM 12751 | -25.237361 | -65.900722 | 18 | 14 | 56.53 | 27.42 | 15.36 | 9.98 | 13.15 | 7.9 |  |  |  |  |  |  |
| Lramirezae | LJAMM 12801 | -25.237361 | -65.900722 | 25 | 16 | 56.02 | 25.58 | 14.61 | 10.42 | 15.98 | 7.05 |  |  |  |  |  |  |
| Lramirezae | LJAMM 12802 | -25.237361 | -65.900722 | 20 | 15 | 52.15 | 22.86 | 14.58 | 9.9 | 15.12 | 7.31 |  |  |  |  |  |  |
| Lramirezae | LJAMM 12803 | -25.237361 | -65.900722 | 22 | 17 | 48.98 | 21.28 | 13.42 | 14.11 | 14.19 | 6.47 |  |  |  |  |  |  |
| Lramirezae | LJAMM 12804 | -25.237361 | -65.900722 | 22 | 15 | 52.73 | 25.72 | 14.14 | 9.83 | 14.77 | 6.64 |  |  |  |  |  |  |
| Lramirezae | LJAMM 12805 | -25.237361 | -65.900722 | 21 | 16 | 51.71 | 25.95 | 13.72 | 8.94 | 14.49 | 7.09 |  |  |  |  |  |  |
| Lramirezae | LJAMM 15680 | -25.236722 | -65.9065 | 22 | 15 | 50.15 | 22.16 | 13.96 | 9.61 | 15.1 | 5.98 |  |  |  |  |  |  |
| Lramirezae | LJAMM 15686 | -25.236722 | -65.9065 | 26 | 17 | 52.37 | 21.72 | 14.22 | 10.37 | 15.53 | 5.97 |  |  |  |  |  |  |
| Lramirezae | LJAMM 15687 | -25.236722 | -65.9065 | 22 | 16 | 52.63 | 22.9 | 14.32 | 9.96 | 15.2 | 6.69 |  |  |  |  |  |  |
| Lramirezae | LJAMM 4416 |  |  |  |  |  |  |  |  |  |  | JN410394 | JN410520 | JN410425 | JN410463 | JN410498? |  |
| Lrobertmertensi | LJAMM 332 | -28.678194 | -65.970917 | 22 | 16 | 53.63 | 23.51 | 14.59 | 10.86 | 16.38 | 7.62 |  |  |  |  |  |  |
| Lrobertmertensi | LJAMM 716 | -28.033333 | -66.15 | 26 | 16 | 52.88 | 24.25 | 14.86 | 10.81 | 16.04 | 7.14 |  |  |  |  |  |  |
| Lrobertmertensi | LJAMM 730 | -27.878464 | -66.211364 | 27 | 18 | 54.21 | 25.31 | 15.68 | 11.3 | 17.25 | 7.53 |  |  |  |  |  |  |
| Lrobertmertensi | LJAMM 2064 | -28.300344 | -67.373533 | 23 | 15 | 59.23 | 28.23 | 15.57 | 11.65 | 17.28 | 7.88 |  |  |  |  |  |  |
| Lrobertmertensi | LJAMM 4754 | -27.958306 | -67.636639 | 25 | 17 | 59.26 | 27.03 | 17.52 | 12.12 | 17.59 | 7.9 |  |  |  |  |  |  |
| Lrobertmertensi | LJAMM 16593 | -27.634194 | -66.171056 | 22 | 15 | 51.77 | 23.67 | 13.89 | 9.98 | 14.73 | 6.48 |  |  |  |  |  |  |
| Lrobertmertensi | FML 01706 | -27.878464 | -66.211364 | 24 | 18 | 64.45 | 28.8 | 17.59 | 11.55 | 17.35 | 7.9 |  |  |  |  |  |  |
| Lrobertmertensi | FML 01706 | -27.878464 | -66.211364 | 22 | 19 | 60.14 | 28 | 17.58 | 11.58 | 17.3 | 7.9 |  |  |  |  |  |  |
| Lrobertmertensi | FML 01847 | -27.781889 | -66.204458 | 21 | 16 | 63.42 | 30.5 | 16 | 11.47 | 17.58 | 7.09 |  |  |  |  |  |  |
| Lrobertmertensi | LJAMM 1961 |  |  |  |  |  |  |  |  |  |  | JN410398?? | JN410535 | JN410421 | JN410458 | JN410493? |  |
| Lsaxatilis | AMNH 126616 | -33.169917 | -65.038306 | 22 | 19 | 44.64 | 22 | 13.27 | 8.73 | 13.47 | 6.21 |  |  |  |  |  |  |
| Lsaxatilis | AMNH 65193 | -33.169917 | -65.038306 | 23 | 15 | 45.51 | 24.65 | 13 | 8.81 | 13.95 | 6.25 |  |  |  |  |  |  |
| Lsaxatilis | AMNH 65195 | -33.169917 | -65.038306 | 22 | 16 | 45.23 | 21.85 | 13.36 | 8.8 | 13.92 | 6.23 |  |  |  |  |  |  |
| Lsaxatilis | AMNH 65196 | -33.169917 | -65.038306 | 21 | 16 | 45.71 | 21.68 | 13.35 | 8.83 | 13.95 | 6.22 |  |  |  |  |  |  |
| Lsaxatilis | SDSU 1736 | -32.8915 | -64.868083 | 23 | 18 | 47.49 | 20.63 | 13.4 | 8.89 | 14.1 | 6.27 |  |  |  |  |  |  |
| Lsaxatilis | LJAMM 5044 |  |  |  |  |  |  |  |  |  |  | JN410365 | JN410553 | JN410427 | JN410465 | JN410500? |  |
| Lpagaburoi | LJAMM 12816 | -26.736455 | -65.784188 | 21 | 16 | 45.69 | 20.5 | 12.1 | 8.07 | 13.4 | 5.31 |  |  |  |  |  |  |
| Lpagaburoi | FML 2746 | -26.736455 | -65.784188 | 21 | 17 | 48.88 | 22.43 | 13.01 | 8.04 | 13.04 | 5.98 |  |  |  |  |  |  |
| Lpagaburoi | FML 2746 | -26.736455 | -65.784188 | 20 | 18 | 52.12 | 22.99 | 14.32 | 8.24 | 11.89 | 5.57 |  |  |  |  |  |  |
| Lpagaburoi | FML 2435 | -26.736455 | -65.784188 | 19 | 17 | 50.78 | 22.68 | 13.5 | 8.17 | 13.11 | 8.02 |  |  |  |  |  |  |
| Lpagaburoi | FML 2435 | -26.736455 | -65.784188 | 20 | 16 | 44.31 | 20.6 | 12.1 | 8 | 12.73 | 5.71 |  |  |  |  |  |  |
| Lpagaburoi | FML 2435 | -26.736455 | -65.784188 | 18 | 16 | 45.94 | 21.86 | 12.4 | 8.01 | 12.76 | 7.19 |  |  |  |  |  |  |
| Lpagaburoi | FML 2435 | -26.736455 | -65.784188 | 19 | 16 | 43.25 | 20.3 | 12.2 | 8 | 12.27 | 7.9 |  |  |  |  |  |  |
| Lpagaburoi | FML 2435 | -26.736455 | -65.784188 | 21 | 18 | 44.8 | 21.29 | 12.3 | 8.02 | 12.89 | 7.05 |  |  |  |  |  |  |
| Lpagaburoi | FML 2633 | -26.736455 | -65.784188 | 19 | 14 | 45.77 | 21.7 | 12.1 | 8.02 | 13.58 | 7.31 |  |  |  |  |  |  |
| Lpagaburoi | FML 2633 | -26.736455 | -65.784188 | 20 | 15 | 47.5 | 21.6 | 12.6 | 8.89 | 13.31 | 6.47 |  |  |  |  |  |  |
| Lpagaburoi | FML 2633 | -26.736455 | -65.784188 | 19 | 16 | 50.93 | 22.5 | 13.04 | 9.61 | 13.38 | 6.64 |  |  |  |  |  |  |
| Lpagaburoi | FML 2633 | -26.736455 | -65.784188 | 19 | 15 | 47.59 | 20.8 | 12.06 | 9.3 | 13.75 | 7.09 |  |  |  |  |  |  |
| Lpagaburoi | FML 2633 | -26.736455 | -65.784188 | 18 | 15 | 42.84 | 19.6 | 11.09 | 7.98 | 12.4 | 5.6 |  |  |  |  |  |  |
| Lpagaburoi | FML 1829 | -26.736455 | -65.784188 | 20 | 16 | 46.06 | 21.2 | 12.06 | 8.53 | 13.64 | 0.21 |  |  |  |  |  |  |
| Lpagaburoi | FML 1829 | -26.736455 | -65.784188 | 20 | 15 | 47.51 | 23 | 12.07 | 8.89 | 14.03 | 0.21 |  |  |  |  |  |  |
| Lpagaburoi | FML N/A | N/A | N/A | N/A | N/A | N/A | N/A | N/A | N/A | N/A | N/A | AY662058 |  |  |  |  |  |
| Lpuna | LJAMM 15646 | -24.139167 | -66.700833 | 20 | 15 | 52.36 | 23.15 | 14.42 | 9.21 | 15.25 | 6.78 |  |  |  |  |  |  |
| Lpuna | LJAMM 15648 | -24.139167 | -66.700833 | 21 | 14 | 44.1 | 19.18 | 13.35 | 8.08 | 14.28 | 5.25 |  |  |  |  |  |  |
| Lpuna | LJAMM 15693 | -24.13925 | -66.701031 | 23 | 15 | 45.32 | 20.81 | 14.09 | 8.99 | 15.62 | 5.6 |  |  |  |  |  |  |
| Lpuna | LJAMM 16068 | -24.13925 | -66.701031 | 24 | 15 | 49.86 | 22.26 | 13.92 | 9.14 | 15.7 | 5.9 |  |  |  |  |  |  |
| Lpuna | LJAMM 16069 | -25.0445 | -66.278333 | 24 | 15 | 43.24 | 19.33 | 13.05 | 8.37 | 14.12 | 5.34 |  |  |  |  |  |  |
| Lpuna | LJAMM 16070 | -25.0445 | -66.278333 | 23 | 17 | 55.91 | 24.94 | 15.43 | 10.05 | 15.98 | 6.37 |  |  |  |  |  |  |
| Lpuna | REE 219 FN | N/A | N/A | N/A | N/A | N/A | N/A | N/A | N/A | N/A | N/A | AY662059 |  |  |  |  |  |
| Lchaltin | LJAMM 12009 | -22.706778 | -65.720111 | 20 | 15 | 60.24 | 29.72 | 16.03 | 11.64 | 16.36 | 7.32 |  |  |  |  |  |  |
| Lchaltin | LJAMM 12010 | -22.706778 | -65.720111 | 22 | 13 | 54.95 | 55.44 | 14.69 | 10.11 | 15.99 | 6.24 |  |  |  |  |  |  |
| Lchaltin | LJAMM 12016 | -22.706778 | -65.720111 | 23 | 15 | 56.13 | 25.46 | 15.06 | 11.21 | 16.18 | 7.04 |  |  |  |  |  |  |
| Lchaltin | LJAMM 12017 | -22.706778 | -65.720111 | 23 | 16 | 56.75 | 26.64 | 15.73 | 10.68 | 16.01 | 6.78 |  |  |  |  |  |  |
| Lchaltin | LJAMM 12018 | -22.706778 | -65.720111 | 27 | 18 | 41.21 | 19.12 | 11.17 | 8.22 | 13.28 | 5.38 |  |  |  |  |  |  |
| Lchaltin | SDSU 3572 | N/A | N/A | N/A | N/A | N/A | N/A | N/A | N/A | N/A | N/A | AY662061 |  |  |  |  |  |
| Lpunmahuida | LJAMM 6458 | N/A | N/A | N/A | N/A | N/A | N/A | N/A | N/A | N/A | N/A | JN410368 | x | JN410432 | JN410470 | JN410505 | x |
| Lwalkeri | MUSM 30816 | N/A | N/A | N/A | N/A | N/A | N/A | N/A | N/A | N/A | N/A | KF92367 | KF923649 |  |  |  |  |
| Lwalkeri | BYU 50342 | N/A | N/A | N/A | N/A | N/A | N/A | N/A | N/A | N/A | N/A | KF923676 | KF923648 |  |  |  |  |
| Lwalkeri | MUSM 31423 | N/A | N/A | N/A | N/A | N/A | N/A | N/A | N/A | N/A | N/A | KF923675 | KF923647 |  |  |  |  |
| Lwalkeri | BYU 50340 | N/A | N/A | N/A | N/A | N/A | N/A | N/A | N/A | N/A | N/A | KF923674 | KF923646 |  |  |  |  |
| Lwalkeri | MUSM 31429 | N/A | N/A | N/A | N/A | N/A | N/A | N/A | N/A | N/A | N/A | KF923673 | KF923645 |  |  |  |  |
| Lwalkeri | BYU 50118 | N/A | N/A | N/A | N/A | N/A | N/A | N/A | N/A | N/A | N/A | KF923672 | KF923644 |  |  |  |  |

**Table S2:** Details of genetic markers used in the current study.

| **Gene** | **Primers** | **Length** | **Type** | **Reference** |
| --- | --- | --- | --- | --- |
| cyt-b | F 5' CCATCCAACATCTCAGCATGATGAAA 3' | 725 | mtDNA | Morando et al. (2003) |
|  | R 5' GGCAAATAGGAARTATCATTC 3' |  |  |  |
| 12S | F 5' GTRCGCTTACCWTGTTACGACT 3' | 883 | mtDNA | Morando et al. (2003) |
|  | R 5' AAAGCACRGCACTGAAGATGC 3' |  |  |  |
| LPB9c | F 5′ TGACTTGTGAGTAGTTAGGGTATGC 3′ | 740 | nDNA | Olave et al. (2011) |
|  | R 5′ TTTGGTGTGGCATGTGCATGTGAAAT 3′ |  |  |  |
| LPB4g | F 5′ TCGAAACTCCTTCAGGGCTA 3′ | 661 | nDNA | Olave et al. (2011) |
|  | R 5′ TTTCCTACCTCGGTCACCAC 3′ |  |  |  |
| LPA11e | F 5′ CAAGGATCCATAGCACAGCA 3′ | 785 | nDNA | Olave et al. (2011) |
|  | R 5′ CACCTTCTGAGGCAATCCAT 3′ |  |  |  |
| LPB11e | F: 5' CAGGAGCTGAGTTCGAGAGG 3' | 823 | nDNA | This study |
|  | R:5' GGAAATGGGGTTTTTGGATT 3' |  |  |  |

**Table S3:** Results of regression of morphological trait data against body size. Degrees of freedom = 142.

| **Response Variable** | | **Coefficient** | | **Estimate** | | **SE** | | **t value** | | **P value** | | **Adjusted R^2^** | | **F statistic** | | **p value** |
| --- | --- | --- | --- | --- | --- | --- | --- | --- | --- | --- | --- | --- | --- | --- | --- | --- |
| Axilla-groin length | | intercept | | -1.57 | | 1.05 | | -1.49 | | 0.14 | | 0.80 | | 575.1 | | < 0.001*** |
| (AG) | | SVL | | 0.49 | | 0.02 | | 23.98 | | < 0.001*** | |  |  |  |  |  |
| Arm length | | intercept | | 1.90 | | 0.59 | | 3.22 | | <0.01** | | 0.75 | | 437 | | < 0.001*** |
| (AL) | | SVL | | 0.24 | | 0.01 | | 20.91 | | < 0.001*** | |  |  |  |  |  |
| Tibia length | | intercept | | -1.48 | | 0.18 | | -8.27 | | < 0.001*** | | 0.75 | | 436 | | < 0.001*** |
| (TbL) | | SVL | | 0.95 | | 0.05 | | 20.88 | | < 0.001*** | |  |  |  |  |  |
| Foot length | | intercept | | -0.01 | | 0.22 | | -0.07 | | 0.95 | | 0.52 | | 156.9 | | < 0.001*** |
| (FL) | | SVL | | 0.69 | | 0.06 | | 12.53 | | < 0.001*** | |  |  |  |  |  |
| Pelvic width | | intercept | | -1.51 | | 0.26 | | -5.74 | | < 0.001*** | | 0.54 | | 167.6 | | < 0.001*** |
| (PW) | | SVL | | 0.87 | | 0.07 | | 12.95 | | < 0.001*** | |  |  |  |  |  |
|  |  |  |  |  |  |  |  |  |  |  |  |  |  |  |  |  |
|  |  | |  | |  | |  | |  | |  |  |  | |  |  |

**Table 4.** Variable loadings for each data type.

**Table S4a:** Variable loadings for each principal component for temperature data.

| **Variable** | **Axis 1** | **Axis 2** | **Axis 3** | **Axis 4** |
| --- | --- | --- | --- | --- |
| Annual mean temp. (B1) | -0.7752 | -0.6273 | -0.0130 | 0.0321 |
| Mean diurnal range (B2) | 0.1594 | -0.2843 | **0.9259** | -0.1894 |
| Isothermality (B3) | 0.7395 | -0.4416 | 0.3447 | -**0.3710** |
| Temp. seasonality (B4) | -0.8206 | 0.5182 | 0.1956 | 0.1209 |
| Max. warm temp.(B5) | **-0.9651** | -0.0397 | 0.2275 | -0.1020 |
| Min. cold temp. (B6) | -0.7626 | -0.0923 | -0.5426 | -**0.3260** |
| Annual temp range (B7) | -0.5831 | 0.1073 | **0.7872** | 0.1661 |
| Mean temp. wet month (B8) | -0.1142 | **-0.9534** | -0.0537 | 0.2460 |
| Mean temp. warm month (B9) | -0.6810 | 0.6633 | 0.0356 | -0.2629 |
| Mean temp. warm quarter (B10) | **-0.9794** | -0.1873 | 0.0386 | 0.0364 |
| Mean temp. cold quater (B11) | -0.5081 | **-0.8347** | -0.1350 | -0.1241 |
|  |  |  |  |  |
| **Proportional Variation** | 49.03% | 27.64% | 18.22% | 4.37% |
| **Cumulative Variation** | 49.03% | 76.67% | 94.89% | 99.26% |
|  |  |  |  |  |
| **Predictor Variables** | B5 | B8 | B2 | B3 |
|  | B10 | B11 | B7 | B6 |

**Table S4b:** Variable loadings for each principal component for precipitation data.

| **Variable** | **Axis 1** | **Axis 2** | **Axis 3** |
| --- | --- | --- | --- |
| Annual prec. (B12) | **-0.9593** | -0.2505 | -0.0745 |
| Prec. wet month (B13) | **-0.9948** | 0.0819 | 0.0529 |
| Prec. dry month (B14) | -0.2322 | -**0.9344** | -0.2539 |
| Prec. seasonality (B15) | -0.7076 | 0.6160 | 0.3114 |
| Prec. wet quarter (B16) | **-0.9945** | 0.0679 | 0.0587 |
| Prec. dry quarter (B17) | -0.1288 | **-0.9716** | -0.1480 |
| Prec. warm month (B18) | -0.4689 | 0.6746 | **-0.5679** |
| Prec. cold month (B19) | -0.3525 | **-0.9041** | 0.2397 |
|  |  |  |  |
| **Proportional Variation** | 47.68% | 44.28% | 7.19% |
| **Cumulative Variation** | 47.68% | 91.96% | 99.15% |
|  |  |  |  |
| **Predictor Variables** | B12 | B14 | B18 |
|  | B13 | B17 |  |
|  | B16 | B19 |  |

**Table S4c:** Variable loadings for each principal component for vegetation data.

| **Variable** | **Axis 1** | **Axis 2** | **Axis 3** |
| --- | --- | --- | --- |
| Mean NDVI | **0.9475** | 0.1261 | **0.2937** |
| Seasonality NDVI | **0.8439** | -0.4631 | **-0.2709** |
| % Tree Cover | 0.2880 | **0.9420** | -0.1725 |
|  |  |  |  |
| **Proportional Variation** | 56.43% | 37.26% | 6.31% |
| **Cumulative Variation** | 56.43% | 93.69% | 100.00% |
|  |  |  |  |
| **Predictor Variables** | Mean NDVI | TreeCov | Mean NDVI |
|  | Seas. NDVI |  | Seas. NDVI |

**Table S5:** Shown are the model of evolution results for environment-phenotype correlations undertaken on 1000 posterior trees. Reported is the proportion of trees supporting each respective model from Brownian Motion (BM), Orstein-Uhlenbeck (OU) and Early Burst (OU) models. Confidence limits (95%) on model weight, AICc and the delta AICs across posterior trees are shown.

| **Dependent Variable** | **Predictor Variable** | **Model** | **Model Weight** | **AICc** | **Delta AICc** |
| --- | --- | --- | --- | --- | --- |
| **Body Shape** | **Vegetation** | **BM (99.81%)** | **0.42 - 0.58** | **-654.16 - -654.08** | **0 - 0** |
|  |  | *OU (0.10%)* | *0.16 - 0.32* | *-653.19 - -652.08* | *0.89 - 2.00* |
|  |  | EB (0.10%) | 0.18 - 0.41 | -654.01 - -652.08 | 0.07 - 2.00 |
|  | **Precipitation** | **BM (99.9%)** | **0.52 - 0.58** | **-630.64 - -629.55** | **0 - 0** |
|  |  | *OU (0%)* | *0.19 - 0.25* | *-628.78 - -627.56* | *1.53 - 2.00* |
|  |  | EB (0.01%) | 0.20 - 0.28 | -628.87 - -627.55 | 1.31 - 2.00 |
|  | **Temperature** | **BM (100%)** | **0.50 - 0.58** | **-570.07 - -570.07** | **0 - 0** |
|  |  | *OU (0%)* | *0.18 - 0.22* | *-568.18 - -568.07* | *1.88 - 2.00* |
|  |  | EB (0%) | 0.21 - 0.32 | -569.18 - -568.07 | 0.88 - 2.00 |
|  | **Elevation** | **BM (99.62%)** | **0.52 - 0.58** | **-765.99 - -765.99** | **0 - 0** |
|  |  | *OU (0.27%)* | *0.19 - 0.24* | *-764.31 - -763.99* | *1.68 - 2.00* |
|  |  | EB (0.10%) | 0.20 - 0.29 | -764.86 - -763.99 | 1.13 - 2.00 |
| **Body Size** | **Vegetation** | BM (0%) | <0.01 - <0.01 | -52.28 - 6.64 | 12.07 - 46.00 |
|  |  | ***OU (100%)*** | ***0.98 - 1.00*** | ***-66.41 - -47.12*** | ***0 - 0*** |
|  |  | EB (0%) | <0.01 - 0.02 | -56.09 - -11.2 | 8.31 - 41.26 |
|  | **Precipitation** | BM (0%) | <0.01 - <0.01 | -44.37 - 8.42 | 18.88 - 60.54 |
|  |  | ***OU (100%)*** | ***1.00 - 1.00*** | ***-65.67 - -51.81*** | ***0 - 0*** |
|  |  | EB (0%) | <0.01 - <0.01 | -48.55 - 3.80 | 14.88 - 54.80 |
|  | **Temperature** | BM (0%) | <0.01 - 0.01 | -52.2 8- -6.64 | 12.18 - 42.39 |
|  |  | ***OU (100%)*** | ***0.98 - 1.00*** | ***-66.47 - -44.74*** | ***0 - 0*** |
|  |  | EB (0%) | <0.01 - 0.02 | -56.09 - -11.62 | 8.35 - 37.25 |
|  | **Elevation** | BM (0%) | <0.01 - 0.01 | -41.87 - 11.21 | 22.86 - 65.68 |
|  |  | ***OU (100%)*** | ***1.00 - 1.00*** | ***-66.74 - -53.55*** | ***0 - 0*** |
|  |  | EB (0%) | <0.01 - 0.01 | -46.18 - 5.86 | 18.59 - 60.40 |

**Table S6:** Results of tests for evolutionary correlations between environmental (vegetation, precipitation, temperature, and elevation) and body shape traits using an OU model and body size using BM model. We provide the mean and 95% confidence intervals (brackets) for the test statistic, and mean and 95% confidence intervals (brackets) for slope significance *P*–values. Parameter estimates are taken from 1,000 randomly drawn trees from the posterior distribution. Trait correlations that are significant are bolded. *P*-value $\leq$ 0.01 **. The proportions of variance described by multivariate axes (i.e., vegetation, temperature, and precipitation) are shown along with the main loaded variables on those axes (see Table S4).

| **Phenotypic**  **Data** | **Environmental Data** | | | | | | | | | | |
| --- | --- | --- | --- | --- | --- | --- | --- | --- | --- | --- | --- |
|  | Vegetation | | | Temperature | | | | Precipitation | | | Elevation |
|  | Av. NDVI  Seas. NDVI  56.43% | Tree Cover  37.26% | Av. NDVI  Seas. NDVI  6.31% | Max Temp.  Warm Temp. 49.03% | Wet Temp.  Cold Temp.  27.64% | Diur. Range  Ann. Range  18.22% | Isotherm.  Min. Temp.  4.37% | Ann. Prec.  Wet Prec.  47.68% | Dry Prec.  Cold Prec  44.28% | Warm Prec.  7.19% |  |
| Body Shape | **0.50**  **(0.50-0.51)** | 0.15  (0.14-0.18) | 0.19  (0.19-0.21) | 0.21  (0.20-0.21) | 0.18  (0.18-0.18) | 0.36  (0.35-0.36) | 0.25  (0.25-0.26) | 0.32  (0.29-0.34) | 0.08  (0.07-0.10) | 0.13  (0.11-0.14) | 0.13  (0.12-0.13) |
| *P-value* | **<0.02***  **(0.02-0.02)**  **100%** | 0.66  (0.57-0.70)  0% | 0.51 (0.45-0.53)  0.2% | 0.51  (0.51-0.53)  0% | 0.60  (0.60-0.60)  0% | 0.14  (0.14-0.16)  0% | 0.37  (0.37-0.37)  0% | 0.19  (0.15-0.23)  0% | 0.89  (0.85-0.92)  0% | 0.76  (0.71-0.80)  0% | 0.70  (0.69-0.74)  0% |
| Body Size | **61.07**  **(0.32-359.21)** | 10.04  (0.01-46.41) | **12.13**  **(0.89-52.06)** | 55.01  (<0.01-0.04) | **38.36**  **(0.65-209.12)** | 8.43  (0.28-22.49) | 2.35  (<0.01-15.10) | 43.21  (0.01-0.02) | 3.23  (<0.01-17.18) | 9.99  (0.01-54.71) | 26.30  (<0.01-164.99) |
| *P-value* | **<0.05***  **(<0.01-0.58)**  **84.7%** | 0.25  (<0.01-0.91)  34.4% | **0.05**  **(<0.01-0.36)**  **75.3%** | 0.26  (<0.01-0.95)  41.7% | **0.03***  **(<0.01-0.43)**  **88.4%)** | 0.11  (<0.01-0.60)  47.6% | 0.45  (<0.01-0.97)  13.0% | 0.25  (<0.01-0.94)  42.7% | 0.48  (<0.01-0.97)  11.7% | 0.21  (<0.01-0.93)  48.8% | 0.27  (<0.01-0.96)  38.5% |

**Figure S1:** Phylogenetic distribution of morphological data variation for each variable used in analyses.

**Figure S1a:** Body size data (log(SVL) distributed across the *L. gracilis* clade including the northern and southern lineages.

**Figure S1b:** Axilla-groin data (as a ratio of SVL) distributed across the *L. gracilis* clade including the northern and southern lineages.

**Figure S1c:** Arm length (as a ratio of SVL) distributed across the *L. gracilis* clade including the northern and southern lineages.

**Figure S1d:** Foot length (as a ratio of SVL) distributed across the *L. gracilis* clade including the northern and southern lineages.

**Figure S1e:** Pelvic width (as a ratio of SVL) distributed across the *L. gracilis* clade including the northern and southern lineages.

**Figure S1f:** Arm length (as a ratio of SVL) distributed across the *L. gracilis* clade including the northern and southern lineages.

**Figure S2:** Phylogenetic distribution of vegetation data variation for each variable used in analyses.

**Figure S2a:** Principal component vegetation axis 1 distributed across the *L. gracilis* clade including the northern and southern lineages.

**Figure S2b:** Principal component vegetation axis 2 distributed across the *L. gracilis* clade including the northern and southern lineages.

**Figure S2c:** Principal component vegetation axis 3 distributed across the *L. gracilis* clade including the northern and southern lineages.

**Figure S3:** Phylogenetic distribution of precipitation data variation for each variable used in analyses.

**Figure S3a:** Principal component precipitation axis 1 distributed across the *L. gracilis* clade including the northern and southern lineages.

**Figure S3b:** Principal component precipitation axis 2 distributed across the *L. gracilis* clade including the northern and southern lineages.

**Figure S3c:** Principal component precipitation axis 3 distributed across the *L. gracilis* clade including the northern and southern lineages.

**Figure S4:** Phylogenetic distribution of temperature data variation for each variable used in analyses.

**Figure S4a:** Principal component temperature axis 1 distributed across the *L. gracilis* clade including the northern and southern lineages.

**Figure S4b:** Principal component temperature axis 2 distributed across the *L. gracilis* clade including the northern and southern lineages.

**Figure S4c:** Principal component temperature axis 3 distributed across the *L. gracilis* clade including the northern and southern lineages.

**Figure S4d:** Principal component temperature axis 4 distributed across the *L. gracilis* clade including the northern and southern lineages.

**Figure S5:** Phylogenetic distribution of elevation data (log transformed) variation for each variable used in analyses.
